# RELSA—A multidimensional procedure for the comparative assessment of well-being and the quantitative determination of severity in experimental procedures

**DOI:** 10.3389/fvets.2022.937711

**Published:** 2022-11-11

**Authors:** Steven R. Talbot, Birgitta Struve, Laura Wassermann, Miriam Heider, Nora Weegh, Tilo Knape, Martine C. J. Hofmann, Andreas von Knethen, Paulin Jirkof, Christine Häger, André Bleich

**Affiliations:** ^1^Institute for Laboratory Animal Science and Central Animal Facility, Hannover Medical School, Hanover, Germany; ^2^Fraunhofer Institute for Translational Medicine and Pharmacology (ITMP), Frankfurt, Germany; ^3^Department of Anaesthesiology, Intensive Care Medicine and Pain & Therapy, University Hospital Frankfurt, Frankfurt, Germany; ^4^Office for Animal Welfare and 3Rs, University of Zurich, Zurich, Switzerland

**Keywords:** severity assessment, laboratory animal, animal welfare, data science, animal experiments, sepsis model, colitis model, surgical models

## Abstract

Good science in translational research requires good animal welfare according to the principles of 3Rs. In many countries, determining animal welfare is a mandatory legal requirement, implying a categorization of animal suffering, traditionally dominated by subjective scorings. However, how such methods can be objectified and refined to compare impairments between animals, subgroups, and animal models remained unclear. Therefore, we developed the RELative Severity Assessment (RELSA) procedure to establish an evidence-based method based on quantitative outcome measures such as body weight, burrowing behavior, heart rate, heart rate variability, temperature, and activity to obtain a relative metric for severity comparisons. The RELSA procedure provided the necessary framework to get severity gradings in TM-implanted mice, yielding four distinct RELSA thresholds L1<0.27, L2<0.59, L3<0.79, and L4<3.45. We show further that severity patterns in the contributing variables are time and model-specific and use this information to obtain contextualized *between* animal-model and subgroup comparisons with the severity of sepsis > surgery > restraint stress > colitis. The bootstrapped 95% confidence intervals reliably show that RELSA estimates are conditionally invariant against missing information but precise in ranking the quantitative severity information to the moderate context of the transmitter-implantation model. In conclusion, we propose the RELSA as a validated tool for an objective, computational approach to comparative and quantitative severity assessment and grading. The RELSA procedure will fundamentally improve animal welfare, data quality, and reproducibility. It is also the first step toward translational risk assessment in biomedical research.

## Introduction

Good science and high-quality data from animal experiments in basic and translational research require good animal welfare. Consequently, researchers are obligated to ensure the best possible welfare of their research animals, in line with the refinement principle in the 3Rs (1, 2). Therefore, the determination of laboratory animal welfare is embedded in many international animal protection guidelines and acts, e.g., the Guide for the Care and Use of Laboratory Animals (3) and the European Directive on the protection of animals used for scientific purposes (4).

Animal welfare describes the status of life quality, which relies on the consideration and promotion of things to achieve good animal welfare (5, 6). Its assessment requires monitoring animal affective states with positive and negative valence (7). The term severity assessment emphasizes categorizing negative affective states in animals, explicitly under experimental conditions. It aims to recognize signs of suffering and is essential for possible interventions to relieve the burden during experiments and promotes the refinement of procedures. Nevertheless, to comply with scientific and regulatory requirements, an accurate, evidence-based severity assessment and the classification of severity conditions are needed (8).

These aims raise the need for a more precise and data-centered evaluation of impaired animals, resulting in a more holistic analysis less influenced by human decision bias. Further, integrating multimodal and multivariate methods requires increased methodological awareness and integration into severity assessment, in general, to ensure, for example, inter-animal-model comparability.

Currently, outcome measures from physiology, biochemistry, clinical sciences, and the behavioral sciences are considered to best capture the welfare state of the animals under experimentation (7). This notion often leads to an assortment of results whose interpretation and categorization remain with the scientist. However, few veterinary studies combine multiple collected variables to provide a more comprehensive method for evaluating animal wellbeing. For example, Principal Component Analysis (PCA) was performed in a study assessing the severity of procedures conducted in three epilepsy models. In this study, multiple behavioral and biochemical parameters were analyzed. The PCA revealed the most informative orthogonal parameters and combined them in a Composite Measures Scheme (CMS) used in comparative severity assessment (9). However, the PCA method can be misleading when data are highly collinear.

In another study investigating the severity of neuroscientific surgeries in rats, a supervised Machine Learning method with a radial Support Vector Machine kernel was used to classify distinct invasive procedures (10), e.g., with the heart rate and activity information as input dimensions. These studies support the concept that incorporating mathematical methods to explain higher-dimensional relationships in the data beyond the traditional scope of simple inferential statistics successfully helps determine states of wellbeing and the severity of experimental procedures (11–13).

Consequently, our study aimed to develop an algorithm-based composite system that provides an objective animal welfare assessment with an arbitrary number of input variables and further introduces the concept of adding relational context to the quantitative severity assessment. With this methodology, and, e.g., using the same measured variables in three independent animal models, we will show that relative severity can be used to compare states of wellbeing between individual animals, treatment groups, and animal models.

Furthermore, this level of comparability is achieved with a collection of physiological, clinical, and behavioral outcome measures from a surgical mouse model, resulting in the development and application of the RElative Severity Assessment (RELSA) procedure. We hypothesize that the individual outcome measures can signal changes in severity in laboratory animals, but they do so at different reporting characteristics over time. Thus, using a weighted composite like the RELSA score helps minimize information loss when variables are missing and allows severity comparisons at different levels, such as the individual or model scale.

The current study is divided into two parts: first, objective parameters were utilized for an actual severity assessment to show the general applicability of RELSA in a well-established method (TM implantation). This surgical model has the advantage of providing a collection of objective variables such as heart rate, heart rate variability, and activity and is officially classified as moderate severity according to the EU directive. In the second part, we aim at the severity grading of different animal models involving inflammation, stress, and sepsis based on identical variables using RELSA. This analysis resulted in an evidence-based severity comparison of experimental procedures, providing scientists and regulators with more precise estimates of severity gradings for experiments and tangible approaches for refinement. Furthermore, these results show that the reference data derived from the surgical model already provide a basis for routine use of this method, e.g., in daily severity monitoring, without the need to gain this data on additional animals. Furthermore, with the RELSA method, researchers are free to define their reference data when needed, as recently shown in a study comparing the severity of genetic, stress-based, and pharmacological depression models (14). The RELSA procedure is, therefore, not bound to an invasive procedure and can be applied as an objective severity measure in a wide range of research models.

## Methods

### Ethical statement

Experiments involving surgery, DSS colitis, and stress were approved by the Local Institutional Animal Care and Research Advisory Committee and permitted by the Lower Saxony State Office for Consumer Protection and Food Safety (LAVES, Oldenburg, Lower Saxony, Germany; license 15/1905). The application for the animal experiments involving sepsis (authorization no. V54–19 c 20/15 - F152/1016) was approved by the local Ethics Committee for Animal Research (Darmstadt, Hessen, Germany). All procedures followed the German animal protection law and the European Directive 2010/63/EU.

### Animals, housing conditions, and husbandry

Female C57BL/6J mice undergoing surgery only (transmitter implantation and Sham groups), DSS colitis, or stress induction were obtained from the Central Animal Facility, Hannover Medical School, Hannover, Germany. For the sepsis study, male C57BL/6N mice were obtained from Charles River Laboratories, Sulzfeld, Germany (for an overview of the studies, mice, and animal numbers, see Supplementary Table 1). The mice were free of the viral, bacterial, and parasitic pathogens listed in the Federation of European Laboratory Animal Science Association (15). A sentinel program monitored their health status throughout the experiments. The mice were housed at the Central Animal Facilities of the MHH (surgery, colitis, stress groups) in macrolon type-II cages (360 cm^2^; Tecniplast, Italy), which were changed once per week. Cages were bedded with autoclaved softwood shavings (poplar wood; AB 368P, AsBe-wood GmbH, Buxtehude, Germany), paper nesting material (AsBe-wood GmbH, Buxtehude, Germany), and two cotton nesting pads (AsBe-wood GmbH, Buxtehude, Germany). Room conditions were standardized (22 ± 1°C; humidity: 50–60%; 14:10 h light/dark cycle). Mice were fed standard rodent food (Altromin 1324, Altromin, Lage, Germany) *ad libitum*, and autoclaved (135°C/60 min) distilled water was provided *ad libitum*. Two female persons handled the mice. For the sepsis experiments, the mice were housed at the animal facility of Fraunhofer IME-TMP, Frankfurt, Germany, in IVC cages (501 cm^2^; GM500, Tecniplast, Italy), which were changed once per week (but never during sepsis). These cages were bedded with softwood shavings (H0234-200, ssniff Spezialdiäten GmbH, Germany), paper nesting material (Sizzlenest, H4201-11, ssniff Spezialdiäten GmbH, Germany) and a mouse igloo (#13100 Plexx BV, Netherlands). Room conditions were standardized (22 ± 2°C; humidity: 45–65%; 12:12 h light/dark cycle including a 30 min twilight phase at the beginning and end of the light/dark phases). The mice were fed standard rodent food (V1534-000, ssniff Spezialdiäten GmbH, Germany) *ad libitum*, and tap water was provided *ad libitum*. Animals were allocated randomly to the testing groups and habituated to the experimental environment before the surgical procedure.

### Transmitter implantation

The mice for the surgery, colitis, and stress studies were 9–10 weeks old. Transmitters (ETA-F10 or HD-X11; DSI, St Paul, MN, USA) were aseptically implanted into the intraperitoneal cavity with electrodes placed subcutaneously for a bipolar lead II configuration under general isoflurane anesthesia. Sham-operated mice underwent aseptic surgery without implantation of the transmitters. General anesthesia was induced in an induction chamber (15 × 10 × 10 cm) with 5 vol% isoflurane (Isofluran CP^®^, CP Pharma, Burgdorf, Germany) and an oxygen flow (100% oxygen) of 6 l/min. After confirming the absence of the righting reflex and removal from the chamber, anesthesia was maintained via an inhalation mask with 1.5–2.5 vol% isoflurane and an oxygen flow of 1 l/min. The corneal reflex was used in combination with the eyelid-closing reflex and the toe pinch reflex to determine the depth of anesthesia. Personnel involved have been trained and were experienced in performing these assays carefully and very softly to omit any damage. The eyes were moistened with eye ointment to protect them from drying out (Bepanthen^®^, Bayer AG, Leverkusen, Germany). After reaching total anesthesia, the surgical area was shaved, and the mice were placed in the surgical field in dorsal recumbency with the head toward the surgeon. During the entire duration of the anesthesia, the mice were placed on a heating pad at 37.0 ± 1.0°C to prevent hypothermia. EMLA^®^ cream (25 mg/g Lidocain + 25 mg/g Prilocain; Aspen Germany GmbH, Munich, Germany) was used for local anesthesia at the incision sites. The mice that underwent only surgery received either preoperative 200 mg/kg metamizole (Novaminsulfon 500 mg Lichtenstein, Zentiva Pharma GmbH, Frankfurt am Main, Germany) subcutaneously (s.c.) and postoperative 200 mg/kg metamizole orally via the drinking water until day 3 or preoperative 5 mg/kg carprofen (Rimadyl, Zoetis Deutschland GmbH, Berlin, Germany) s.c. and postoperative 2.5 mg/kg s.c. every 12 h until day 3. The mice that underwent additional colitis or stress induction were treated using the metamizole analgesia regimen.

In the CLP study, mice aged 12–14 weeks were anesthetized via s.c. injection of 120 mg/kg in 10 ml/kg ketamine (Ketaset^®^, Zoetis Deutschland GmbH, Berlin, Germany) and 8 mg/kg in 10 ml/kg xylazine (Rompun^®^, Bayer Vital GmbH, Leverkusen, Germany). Perioperative management was the same as described above. The blood pressure catheter was placed in the left carotid artery and positioned so that the gel-filled sensing region of the catheter was ~2 mm in the aortic arch. The telemetry transmitter was placed along the lateral flank between the forelimb and hindlimb, close to the back midline. Biopotential ECG leads were tunneled subcutaneously to achieve positioning analogous to lead II in human ECG. For postsurgical analgesia, 200 mg/kg metamizole s.c. (Novaminsulfon 1,000 mg Lichtenstein, Zentiva Pharma GmbH, Frankfurt/Main, Germany) was administered at the first signs of waking up. For postsurgical analgesia, 5 mg/kg carprofen (Rimadyl, Zoetis Deutschland GmbH, Berlin, Germany) was administered s.c. on the evening of the day of the surgery and the morning and evening of day 1 and day 2 after surgery. After fully recovering from the anesthesia, mice were put back into their home cage, and the continuous data acquisition of all physiological parameters began immediately. Mice were randomly allocated to the testing groups and habituated to the experimental environment before the surgical CLP or CLP Sham procedure.

### Sham surgery

Sham-operated mice were used as controls for assessing the severity of transmitter implantation and underwent an aseptic laparotomy without transmitter implantation (Sham mice or animals) under the same conditions as described above (surgery studies), including anesthetic and analgesic regimens.

### Burrowing behavior

One week before intraperitoneal transmitter implantation or the corresponding Sham surgery, the mice were housed pairwise in type ll macrolon cages filled with aspen bedding material (AsBewood GmbH, Buxtehude, Germany) and two compressed cotton nesting pads (AsBewood GmbH, Buxtehude, Germany). On days five and four before surgery, the burrowing apparatus was provided to the animals to train them in the burrowing behavior (16). Baseline measurements were taken on days two and one before surgery. A 250 mL plastic bottle with a length of 15 cm, a diameter of 5.5 cm, and a port diameter of 4 cm was used as a burrowing apparatus. It was filled with 140 ± 1.5 g of the standard diet pellets of the mice (Altromin1324, Lage, Germany). For burrowing testing after surgeries (1st, 2nd, 3rd, 5th, and 7th night after surgery), mice were singly housed in a type-II macrolon cage with autoclaved hardwood shavings. The burrowing bottles were placed in the left corner. Half of the used nesting material from the home cage was provided as a shelter in the right corner. The tests started 3 h before the dark phase. The bottles containing the remaining pellets were placed back into the cages and weighed the following day (burON, “overnight burrowing performance”) again.

### Cecal ligation and puncture surgery

At the earliest 6 days or after reestablishing a regular circadian rhythm after the surgical implantation of the telemetry transmitter device, male C57BL/6JN mice were used for the CLP experiments. The CLP surgery and the subsequent start of the experiments were conducted in the morning to control circadian variations. The mice were weighed, and 30 min before surgery, 0.05 mg/kg buprenorphine was injected s.c. (Bupresol^®^ 0.3 mg/ml, CP-Pharma HmbH, Burgdorf, Germany). The mice were anesthetized using isoflurane (2–3% Forene^®^, AbbVie Deutschland GmbH & Co. KG, Wiesbaden, Germany) and placed on their back on a heating pad while continuously connected to the isoflurane anesthesia. The eyes were moistened with eye ointment. Xylocaine (Xylocain^®^ Pumpspray Dental, AstraZeneca GmbH, Wedel, Germany) was used for local anesthesia using two puffs of a ready-to-use pump spray containing 10 mg lidocaine per puff at the incision site. The corneal reflex was used in combination with the eyelid-closing reflex and the toe pinch reflex to determine the depth of anesthesia. Personal involved have been trained and were experienced in performing these assays carefully and very softly to omit any damage. During the entire period of anesthesia, the mice were on a heating pad at 37.0 ± 1.0°C. The abdominal cavity was aseptically opened via a midline laparotomy incision of approximately 3 cm, and the cecum was exposed. Subsequently, the cecum was 2/3 ligated (Nylon Monofilament Suture 6/0, Fine Science Tools GmbH, Heidelberg, Germany) distal to the ileocecal valve, while care was taken that the intestinal continuity was maintained. The exposed cecum was punctured twice, “through-and-through,” with a 21-gauge needle. Next, sufficient pressure was applied to the cecum to extrude fecal material from each puncture site (~ 1 mm). The cecum was returned to the abdominal cavity and placed in the upper central abdomen. Following this procedure, the peritoneum was closed with three-knot fissures with non-resorbable sterile suture material (Nylon Monofilament Suture 7/0, Fine Science Tools GmbH, Heidelberg, Germany), and the upper skin layer was stapled with sterile clips (Michel Suture Clips 7.5 × 1.75 mm, Fine Science Tools GmbH, Heidelberg, Germany). For the mice undergoing a Sham laparotomy, the same procedure was performed without CLP. After fully recovering from the anesthesia, the mice were put back into their home cage, after which the continuous data acquisition of all physiological parameters began immediately. The mice received 0.1 mg/kg buprenorphine s.c. 3 h after surgery and subsequently every 8 h for the rest of the experiment. At the end of the experiments, mice were anesthetized deeply with isoflurane and killed by cervical dislocation.

### Colitis induction and restraint stress

After intraperitoneal transmitter implantation and 28 days of postoperative recovery, the female C57BL6/J mice were exposed to 0% (control; receiving water only) or 1% DSS (colitis; mol wt 36,000–50,000; MP Biomedicals, Eschwege, Germany) in drinking water for 5 consecutive days to induce intestinal inflammation. The mice were weighed daily, and the telemetry-derived parameters were recorded: hr, hrv, activity, and temperature. The third group of mice was subjected to restraint stress (colitis + stress) and DSS treatment. The mice were inserted into restraint tubes on 10 consecutive days (d1-d10) for 60 min (from 09:00 to 10:00 a.m.). The restraint tubes (23-mm internal diameter, 93-mm length) consisted of transparent acrylic glass with ventilation holes (8 mm diameter) and a whole distance spanning 7-mm–wide opening along the upper side of the tube. The ends of the tubes were sealed on one side by a piece of acrylic glass with a slot for the animals' tail and the other side by a fixed solid plastic ring. The mice could rotate around their axis but could not move horizontally.

### Data characterization and RELSA pre-processing

Data were brought into the tabular format required for RELSA analysis (Supplementary material 2). Up to six outcome measures were used in the calculations (body weight change [bwc], burrowing overnight [burON], heart rate [hr], heart rate variability [hrv], body temperature [temp], and activity [act]). Five parameters were used in the animal model comparisons because burON was not determined in all included studies. The RELSA pre-processing was initiated with the normalization process. The quantitative data were normalized to the range [0;100]% with 100% as starting values [e.g., based on physiological or baseline conditions, e.g., on pre-surgery day (−1), Supplementary material 2 for an example].

### The RELSA methodology required a reference set

**Figure 1:**
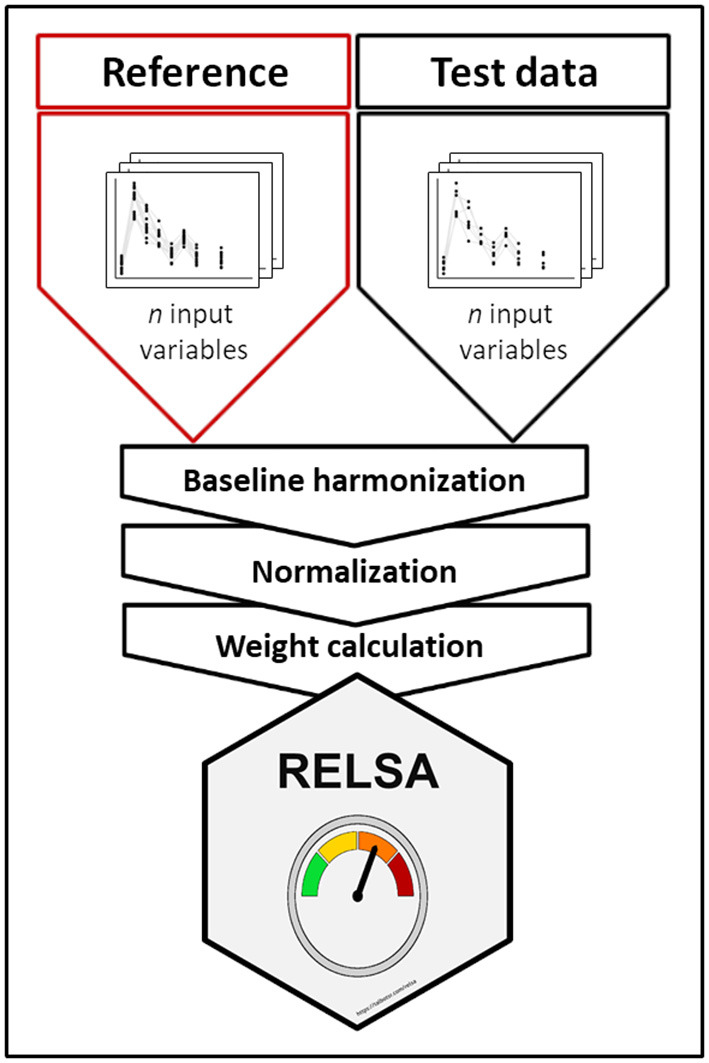
Graphical representation of the RELSA procedure. RELSA requires a reference set that provides qualitative severity context to the quantitative RELSA comparisons. Thus, independent test data can be related to the reference set, enabling an objective relative severity grading of individual animals, subgroups, and models. Before the data can enter the RELSA procedure, the pipeline requires a baseline harmonization so that changes are always relative to a starting point. The normalization to the baseline enables the calculation of the RELSA weights (RW), which are expressions of similarity to the reference set and must be defined with a fixed number of input variables. Later, any number of these variables in the test data can be quantitatively referenced.

Therefore, the surgery model was defined as the RELSA reference set. According to Annex XIII of the EU directive, surgical interventions under general anesthesia, such as TM implantations or Sham surgeries, are categorized as “moderate” in terms of severity. Since the subsequent RELSA analyses were referenced to the values in the reference set (self-reference is possible), they also obtained a relative qualitative severity level. More information on the RELSA procedure is available in Supplementary material 3. A graphical representation of the RELSA procedure is shown in Figure 1. Further, in Supplemental material 4, a simple explanation of the RELSA is demonstrated with examples.

At each observed point in time (t), differences to the normalized baseline in each contributing outcome measure (i) were calculated. Then, to establish the quantitative severity context, the differences were divided by the normalized maximum-reached differences in the respective variables in the reference set. This operation yielded the RELSA weights (RW, see formula 1). Again, care was taken to include the direction of unfolding severity in each outcome measure (e.g., impairments decreased bwc but heightened hr in the included models). The RW were expressions of similarity concerning the maximum-reached value observed in the reference set at any observed point in time. This step also regularized differences in variable contributions at any given severity level, especially in highly collinear contributors.

Larger differences were given more weight, and the final RELSA score was calculated by the root mean square (RMS) of the available RW divided by the number of variables (N) (see formula 2). Missing variables did not contribute to the RELSA, whereas values equal to or above baseline level contributed zero. Furthermore, levels of severity in the reference data were calculated using the *k*-means algorithm (11). The number of clusters was determined heuristically with a Scree plot (Supplementary Figure 5A). A RELSA value of 1 meant that all contributing variables in a test animal reached the same values as the largest observed deviations in the reference set at the defined severity level (here, “moderate”).


(1) $$R_{w}{}_{i}(t)=\frac{\left(\left|100-i\right|\right)}{\left(\left|100-\max_{i,ref}\right|\right)}\;$$

(2) $$\begin{array}{l} RELSA\left(t\right)=\sqrt{\frac{\sum_{1}^{i}{R_{w,i}}^{2}}{N}}\; \end{array}$$

### Statistics

Data were tested against the hypothesis of normality using the Shapiro-Wilk test. In the case of a rejected Null hypothesis, non-parametric methods were used for group comparisons (e.g., the Kruskal-Wallis and Mann-Whitney U-test). When the assumptions of normal distribution were met, parametric analyses were performed, e.g., with an analysis of variance (ANOVA) and reported with a type III error structure due to the presence of interactions. Singular group comparisons were analyzed with the *t*-test (plus Welch's correction in cases of unequal variances). Multiple comparisons were adjusted with the Tukey-Kramer *post-hoc* test. Comparisons to the baseline were calculated with an ANOVA using a control group, followed by Dunnett's posthoc test. The RELSA_max_ and cluster centroids were bootstrapped 10,000-fold to yield estimates as well as 95% bias-corrected and accelerated (BCa) confidence intervals. With either method, the resulting p-values were considered to be significant at the following levels: 0.05 (^*^), 0.01 (^**^), 0.001 (^***^), and 0.0001 (^****^).

### Software, R-packages, and raw data availability

RELSA was developed in R (version 4.0.3). The following packages were used for analysis and visualization: ggplot2, factoextra, effsize, plyr, emmeans, car, and boot. In addition, radar charts were realized using the fsmb package. The RELSA algorithm and the raw data are available as an R package with complete documentation on GitHub: https://github.com/mytalbot/relsa. The RELSA procedure can also be tested with a limited set of variables in a stand-alone web application: https://calliope.shinyapps.io/RELSAapp/. Finally, raw data are available as text files in the following location: https://github.com/mytalbot/RELSA/tree/master/raw_data.

## Results

### Severity assessment after surgery using single outcome measures

**Figure 2:**
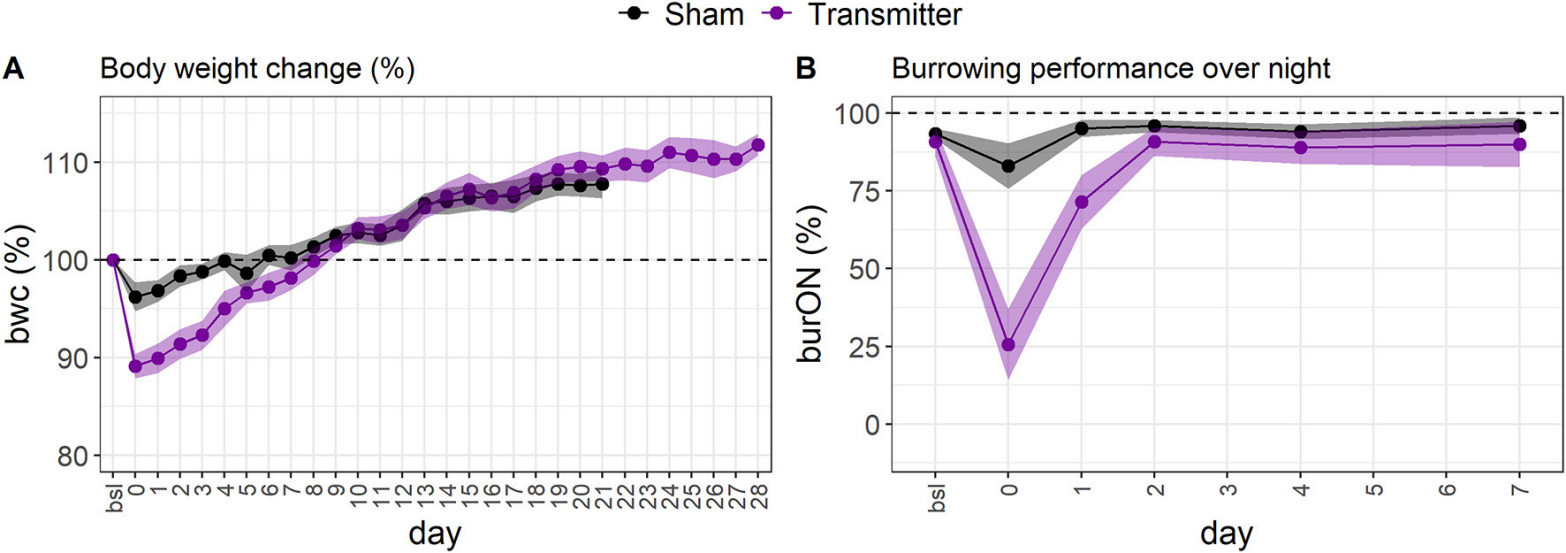
**(A)** The standardized, average body weight change indicates impairments to animal welfare in female C57BL6/J mice. The loss in bwc is most extensive after surgery (day 0) in the TM-implanted group (mauve, *n* = 13). The loss in body weight is less severe in the Sham group (black dots, *n* = 15). On average, the animals recovered back to baseline levels at around day eight. After day eight, the animals gained weight. The errors are depicted as 95% confidence bands. **(B)** The standardized, average overnight burrowing performance (burON) in female C57BL6/J mice also showed a sharp drop after surgery (day 0). However, the performance loss is higher in the TM group (*n* = 13) than in the Sham group (*n* = 15). The burrowing behavior returns faster back to baseline levels than the bwc variable. The animals regained normal burrowing behavior on day 2.

**Figure 3:**
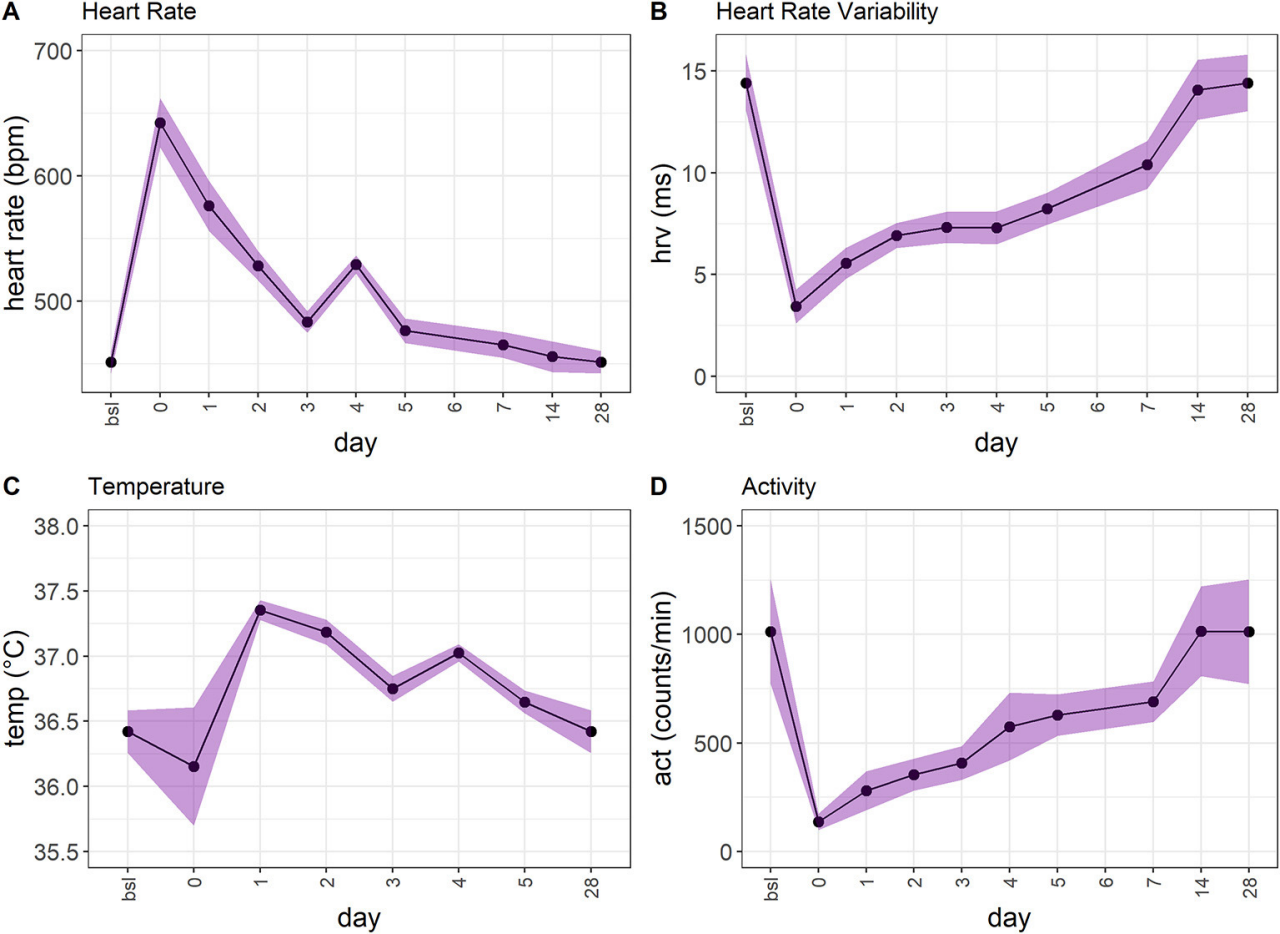
**(A)** The heart rate (hr) in TM-implanted female C57BL6/J mice (*n* = 13) shows an increase on surgery day and returns back to baseline levels. However, the recovery is much slower than, e.g., in burOn and bwc. **(B)** The heart rate variability (hrv) shows a similar development to hr. The maximum drop occurs after surgery (day 0). After that, the animals recover over 28 days. **(C)** The temperature shows an ambiguous development. There is a slight drop after surgery, followed by an increase that slowly returns to baseline levels. However, the range of the temperature variable is small ([34.10; 37.52]°C). **(D)** The activity (act) variable shows a drop after surgery (day 0) and returns back to baseline levels over the next 14 days.

We utilized the surgical model of TM implantation to generate well-defined variables that first can be used as single outcome measures to characterize the model itself (this section) and later for developing and validating the RELSA algorithm (following sections), also providing a reference for assessment of further mouse models. After transmitter (TM) implantation or the corresponding Sham surgeries, we monitored the animals' relative body weight change (bwc) as one of the most frequently used clinical parameters (Figure 2A). TM animals showed an average loss of 10.89% (SD = 2.29%) in body weight on the day of surgery (day 0), which was significantly (*p* < 0.0001) higher than Sham animals which showed only an average loss of 3.78% (SD = 2.93%) which was also significant (*p* = 0.002). No animal lost more than 15.45% in body weight. The bwc values in TM mice returned to baseline levels on day ten after surgery and in Sham animals as early as day six after surgery. Both treatment groups showed weight gain in the progress of the experiment. Further, we observed the overnight burrowing performance (burON). The burrowing also dropped compared to initial values, down to an average of 25.64% (SD = 21.13%). However, in the Sham animals, the loss in burrowing performance was less prominent at an average of 83% (SD = 14.34%) (Figure 2B). The burrowing parameter showed regular burrowing activity on day 2 – two days after surgery. The difference between Sham and TM animals on surgery day was significant (*p* < 0.0001). From the implanted transmitters, additional variables were obtained. The heart rate (hr) spiked after surgery (day 0) at an average of 642.25 (*SD* = 36.12) bpm and returned to baseline levels on day 7 (Figure 3A). The heart rate variability (hrv) showed decreased values after surgery. Its average was lowered to 3.45 (*SD* = 1.52) ms (Figure 3B), corresponding to a 76.1% drop in values. The animals recovered back to baseline on day 14. The body core temperature was measured in a small range ([34.10; 37.52]°C) and showed ambiguous results. After surgery on day 0, there was a slight drop of 0.73% in temp (−0.26°C), followed by an increase on day 1 to 37.35 (SD = 0.14)°C, again followed by a recovery back to baseline levels on day 7 [36.51 (*SD* = 0.17)°C, Figure 3C]. The general activity was reduced after surgery, dropping from an average of 1011.98 (*SD* = 443.14) counts/min to 137.04 (*SD* = 68.87) counts/min. The activity parameter showed recovery to baseline levels on day 14 (Figure 3D). The Supplementary material 6 shows additional inferential statistics on the day-to-baseline and *between* treatment contrasts of the single variables used to indicate changes in severity. Further, the animals were evaluated daily using a clinical score ranging from 0 (no impairment) to 6 (severe impairment) comprising the body weight, the visual evaluation of activity, general health condition, and behavior. An increase to score 2.15 (CI_95%_[1.93; 2.38]) was observed on day 0 (post-op) and returned back to pre-op level on day 9 post-op (Supplementary material 7.1).

This surgery model served as the reference model for the subsequent RELSA development and its validation. The raw data of this and the other animal models with their individual set of variables are available under the following link: https://github.com/mytalbot/RELSA/tree/master/raw_data.

### Severity assessment after surgery using multiple outcome measures in the composite RELSA score

**Figure 4:**
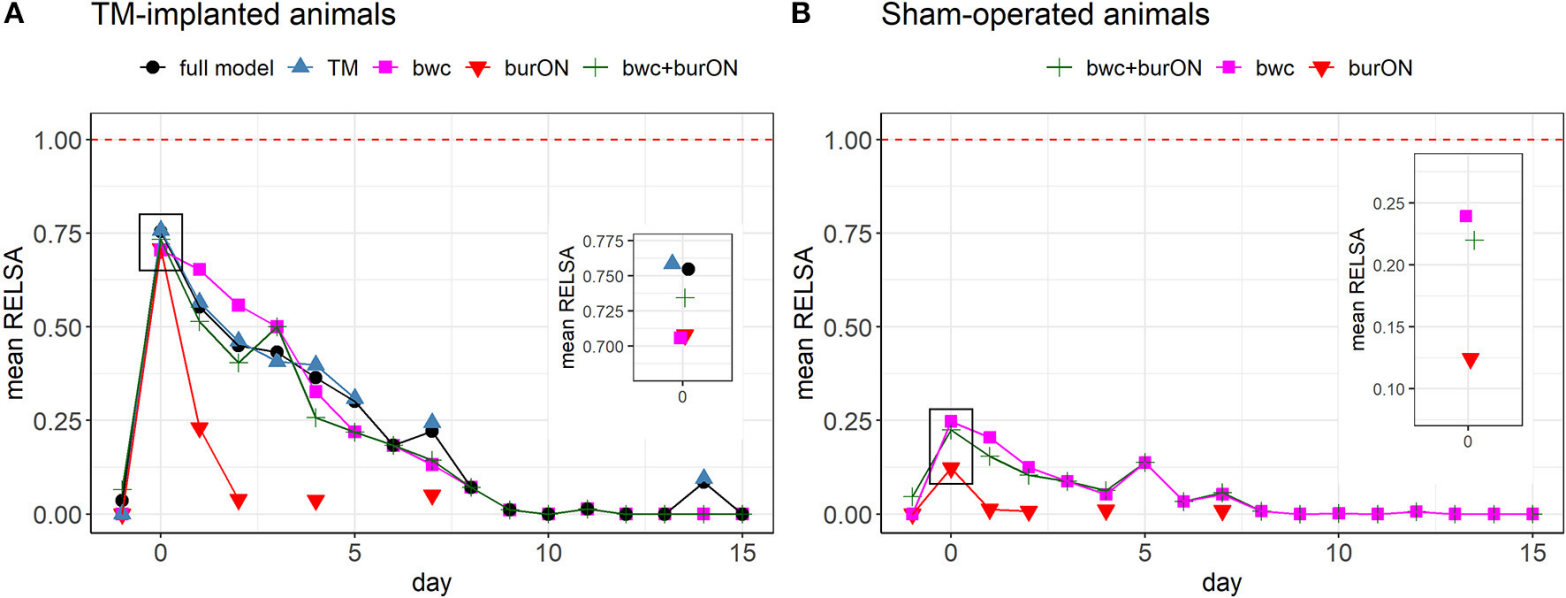
**(A)** Comparison of RELSA performances using different input variables in the TM-implanted animals (*n* = 13). The red dashed line (RELSA = 1) represents the maximum in the reference model. The full model comprised the outcome measures bwc, burON, hr, hrv, act, and temp. The inlay plot focuses on the RELSA values on day 0 (after surgery). Here, the average RELSA values are highest and close together RELSA_mean,0_ = 0.73 (SD = 0.02, range = [0.71; 0.76]) at a low error rate. The highest-performing models are the TM outcomes (hr, hrv, act, and temp, mean = 0.76), followed by the full model (mean = 0.75). During recovery, the performances vary due to changing contributions to the RELSA score. However, the models appear interchangeable as the RELSA weighs and regularizes missing information, especially in highly collinear data. **(B)** Comparison of RELSA performances using different input variables in the Sham-operated animals (*n* = 15). The maximum RELSA is indicated by bwc (mean= 0.24). Note that burON alone has a lower RELSA value than bwc on day 0 (mean = 0.12). However, the combination of bwc and burON can capture most of the severity information on day 0 as indicated by bwc (see inlay plot). Consequently, the combination of bwc and burON (mean= 0.22) performs slightly lower than bwc alone. The burrowing behavior was not measured on all days, so the main RELSA information is dependent on bwc in these cases. The RELSA is relative invariant against missing input variables when multiple measures are included.

While single variables showed differences, e.g., in recovery times (hrv [day 14] vs. burrowing [day 2]) and escalation magnitudes (e.g., a maximum loss in burON of 0%, and a maximum increase of hr at 688.18 bpm), it remained unclear what this contradicting information meant in the context of severity assessment. Therefore, we analyzed how the severity information developed when different variables were combined in the RELSA. As such, the full model (bwc, burON, hr, hrv, temp, and act) was plotted against the TM variables (hr, hrv, temp, and act), the body weight change (bwc), and the burrowing performance overnight (burON), and the body weight change plus the burrowing parameter (bwc+burON) in the TM animals (Figure 4A). Here, the point of maximum severity in the animals was identified as the peak in all models at day 0 (after surgery). The full model showed a mean RELSA score of RELSA_full,0_= 0.75 (*SD* = 0.05), the TM model RELSA_TM,0_= 0.76 (*SD* = 0.06), the bwc RELSA_bwc,0_= 0.71 (*SD* = 0.15), the burON RELSA_burON,0_= 0.71 (*SD* = 0.24), and bwc+burON RELSA_bwc+burON,0_= 0.73 (*SD* = 0.15)(see inlay plot in Figure 4A). On day 0, neither the TM group [*F*_(4, 60)_ = 0.36, *p* = 0.84], nor the Sham group [*F*_(2, 42)_ = 2.69, *p* = 0.08] showed differences in RELSA performances. The exemplary variable permutations reached a mean of RELSA_mean,0_= 0.73 at a high level of precision with the 95% confidence interval in the range CI_95%_[0.71; 0.76]. Therefore, the maximum RELSA score was relatively invariant against small changes in singular variables. This result was corroborated by analyzing the Sham animals, where bwc, burON, and their combination were analyzed using the RELSA. The animals not only showed lower severities than the TM animals with RELSA_bwc+burON,0_= 0.22 (SD= 0.15), RELSA_bwc,0_= 0.25 (*SD* = 0.19), and RELSA_burON,0_= 0.12 (*SD* = 0.13) but also that the RELSA incorporated the short-term spiking of the burON variable on day 0 (Figure 4B) by lowering the average on day zero in the combined model as a consequence of the weighting in the RELSA formula. The difference between the averages of RELSA_bwc+burON,0_ and RELSA_bwc,0_= 0.25 was Δ_RELSA_= 0.03. Consequently, the average RELSA in the Sham group showed lower precision RELSA_Sham,0_= 0.12 (CI_95%_[0.03; 0.36]) than the models above with more contributing variables.

These results show that RELSA enables detection of severity after TM implantation, discriminates different treatments (here: TM implantation vs. Sham operation) and is robust toward the selection of variables. To validate the RELSA performance, we used the clinical score data. The clinical score correlated highly with the RELSA (*r* = 0.98, CI_95%_[0.95; 0.99], *t* = 22.81, *df* = 27, *p* < 0.0001), thereby validating the algorithm. Details on the validation are shown in Supplementary material 7.

### The comparison of severity in individual animals and experimental subgroups can be achieved with the RELSA score and the RELSA_max_ Value

**Figure 5:**
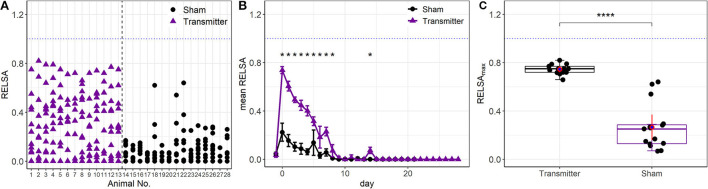
**(A)** Single animal analysis with the full RELSA model (bwc, burON, hr, hrv, act, and temp). The average RELSA score was higher in the TM group than in the Sham group (average RELSA_max,TM_ = 0.75 vs. average RELSA_max,Sham_ = 0.26). In addition, at least three animals in the Sham group have high severity (RELSA>0.4). These animals showed higher losses in bwc and burON than the other animals in the Sham group and were correctly identified. **(B)** Animals in the time-resolved RELSA curves of the Sham and TM groups show a significant treatment:day interaction [*F*_(1, 29)_ = 59.78, *p* > 0.0001]. The subsequent *post-hoc* tests indicate significant differences between treatments (*p < 0.05). On day 14, small spikes in hrv and act unrelated to the surgery occurred and were detected with the RELSA. **(C)** There is a general between-groups difference in severity (RELSA_max_) concerning the TM-implanted (*n* = 13) and Sham animals [*n* = 15, *t*_(26)_ = 8.9, *p* < 0.0001****].

The RELSA procedure was calculated with six variables in the TM group (bwc, burON, hr, hrv, temp, and act) and two in the Sham group (bwc, burON). On day 0, there were no *between*-model differences in the RELSA score due to the high collinearity of the contributing variables (see Figures 4A,B). Subsequently, the individual RELSA scores in the TM animals reached higher values than the Sham animals (Figure 5A). However, at least three animals in the Sham group showed severity anomalies (e.g., RELSA >0.4). These animals were identified as animals 18, 21, and 22 (Figure 5A). Therefore, the RELSA outcome was used to identify the source of these higher severities. The analysis showed that animal 18 had lower values in both outcomes, bwc (89.36%) and burON (46.51%), on day zero, while animals 21 and 22 showed only lowered bwc on day 5 (91.70% and 90.1%) compared to the other Sham animals, e.g., displaying a mean bwc value of 93.02% on day 0. Furthermore, the average RELSA score indicated the higher general severity of the TM-animals at RELSA_TM,0_= 0.73 (*SD* = 0.05) compared to the Sham animals with RELSA_Sham,0_=0.22 (*SD* = 0.15). In addition, the treatment:day interaction was significant in an ANOVA [*F*_(1, 29)_ = 59.78, *p* > 0.0001], and the subsequent *post-hoc* tests showed significant differences between Sham and TM animals on the days 0–8 (*p* < 0.0001) and 14 (*p* < 0.0018) (Figure 5B). The maximum RELSA values from the individual animals were combined into the RELSA_max_ value and used in a subsequent *between*-subgroups comparison. The analysis showed that the highest achieved severity from an integrated set of six variables was significantly different in TM and Sham animals [*t*_(26)_ = 8.9, *p* < 0.0001, Figure 5C].

### The clustering of RELSA_max_ values revealed objective severity levels

In addition to the data for building the RELSA reference set from TM-implanted mice and showing the possible comparisons *between* individual animals and experimental subgroups, we further explored the RELSA as a tool for severity comparisons *between* different animal models. We included three additional animal studies (colitis, stress, and sepsis), with data available on five outcome values (bwc, hr, hrv, temp, and act). Each study was analyzed using the RELSA methodology and was, therefore, referenced against the data from the TM-implanted mice. This quantitative referencing provided the necessary framework for grading the severity information. In addition, we used the individual RELSA_max_ values, as previously described, to map the maximum achieved severity of each animal in each study against the RELSA reference set. This allowed classification of severity levels based on the standardized values from each study. With these data, *k*-means clustering was used to segment the ordered univariate RELSA_max_ outputs into distinct clusters.

First, we estimated the number of clusters to *k* = 4 using Scree analysis. The heuristics of this selection process are shown in the Supplementary Figures 5A,B. The resulting limits of the clustering are shown as dashed lines in Supplementary Figures 5B, 8. The four RELSA_max_ cluster thresholds were L1<0.27, L2<0.59, L3<0.79, and L4<3.45.

**Figure 6:**
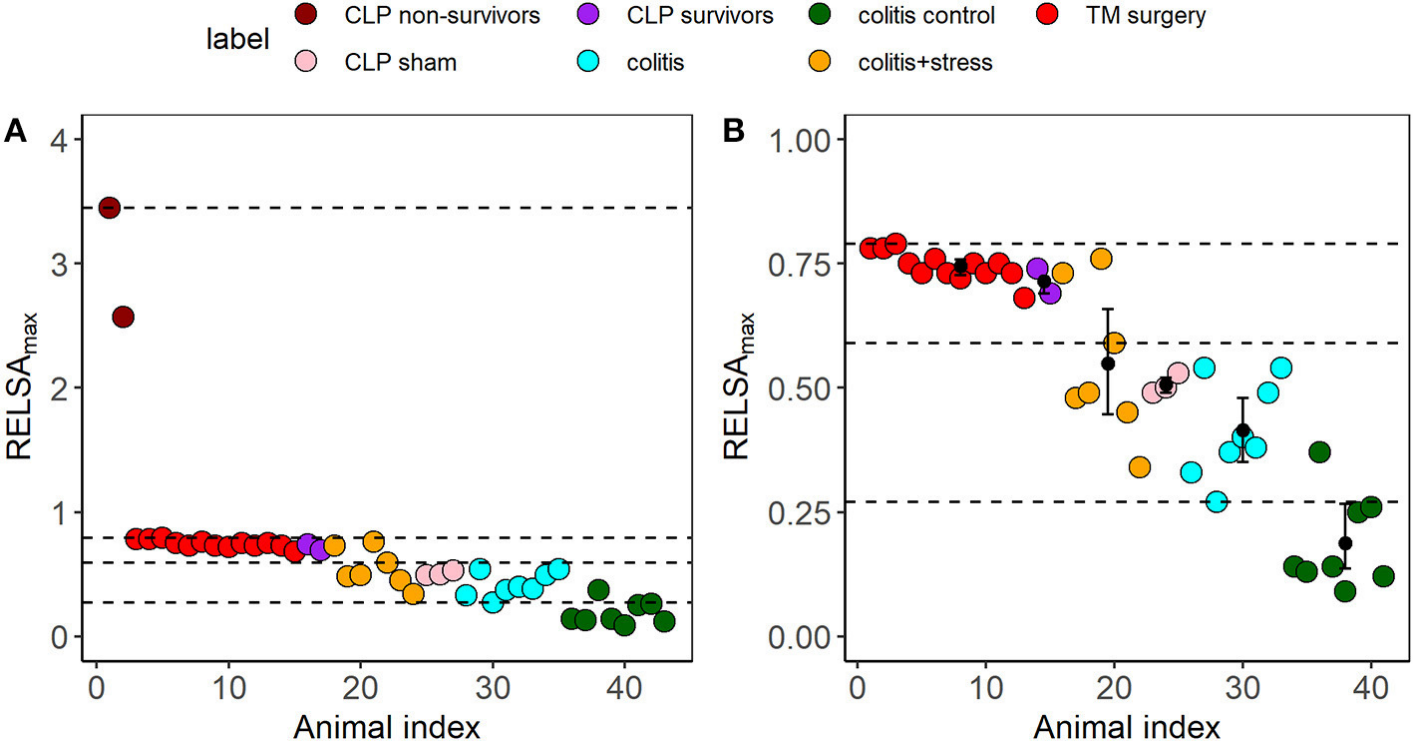
**(A)** Cluster analysis and severity categorization of six distinct subgroups from the three independent animal studies cecal ligation puncture (CLP, sepsis), surgery (TM implantation), and colitis/restraint stress using the RELSA_max_ as the maximum experienced severity information. Each dot represents an animal. In all subgroups, the outcome measures bwc, hr, hrv, temp, and act were used to calculate the RELSA_max_. The dashed lines represent the four severity thresholds from a k-means clustering (L1<0.27, L2<0.59, L3<0.79, and L4<3.45) to enable a comparative grading and categorization of the models and animals. The highest severity was reached by the two CLP non-survivors (RELSAmax>2.5). **(B)** The bootstrapped cluster centers with 95% confidence intervals show that except for the colitis + stress model data, the 95% CIs remain within the identified cluster levels, indicating highly stable severity estimates. Individual animals in the colitis control group showed increased severity due to a drop in activity. Note that the RELSA scale focuses on the range RELSA[0;1]; therefore, the two CLP non-survivors are not visible.

Second, we analyzed and compared the additional studies in terms of severity, using the cluster levels to attribute severity gradings. The other data included mice suffering from colitis induced by dextran sulfate sodium (DSS) and colitis plus additional stress (colitis+stress). In the latter group, the animals received DSS and were subjected to immobilization stress for 1 h on ten consecutive days. The corresponding colitis control animals were treated with water only. Furthermore, we refined data from a study on cecal ligation puncture (CLP) surgery for sepsis induction and the corresponding Sham-operated animals (CLP Sham). Here, the data were divided into CLP survivors and non-survivors. The cluster analysis revealed the highest severity level in CLP non-survivors, followed by a cluster of TM-implanted animals (which comprised the RELSA reference set), followed by CLP survivors. Data from the colitis+stress and colitis study formed the lower severity clusters and CLP Sham-operated animals. Colitis control animals were allocated to the lowest severity cluster (Figure 6).

Furthermore, we investigated how stable the RELSA_max_ distributions were in their group estimates and cluster positions. Some studies or subgroups involved small sample sizes (Supplementary material 1). Therefore, we applied 10,000-fold bootstrapping to assess the 95% confidence intervals of the RELSA_max_ centroids. Except for the colitis + stress study, the confidence intervals remained within their relative *k*-means cluster levels. The confidence interval of the colitis control group did not overlap with any other higher-level confidence interval and did not cross the L1 cluster threshold.

### RELSA generalized model-specific changes in outcome patterns into global severity information

**Figure 7:**
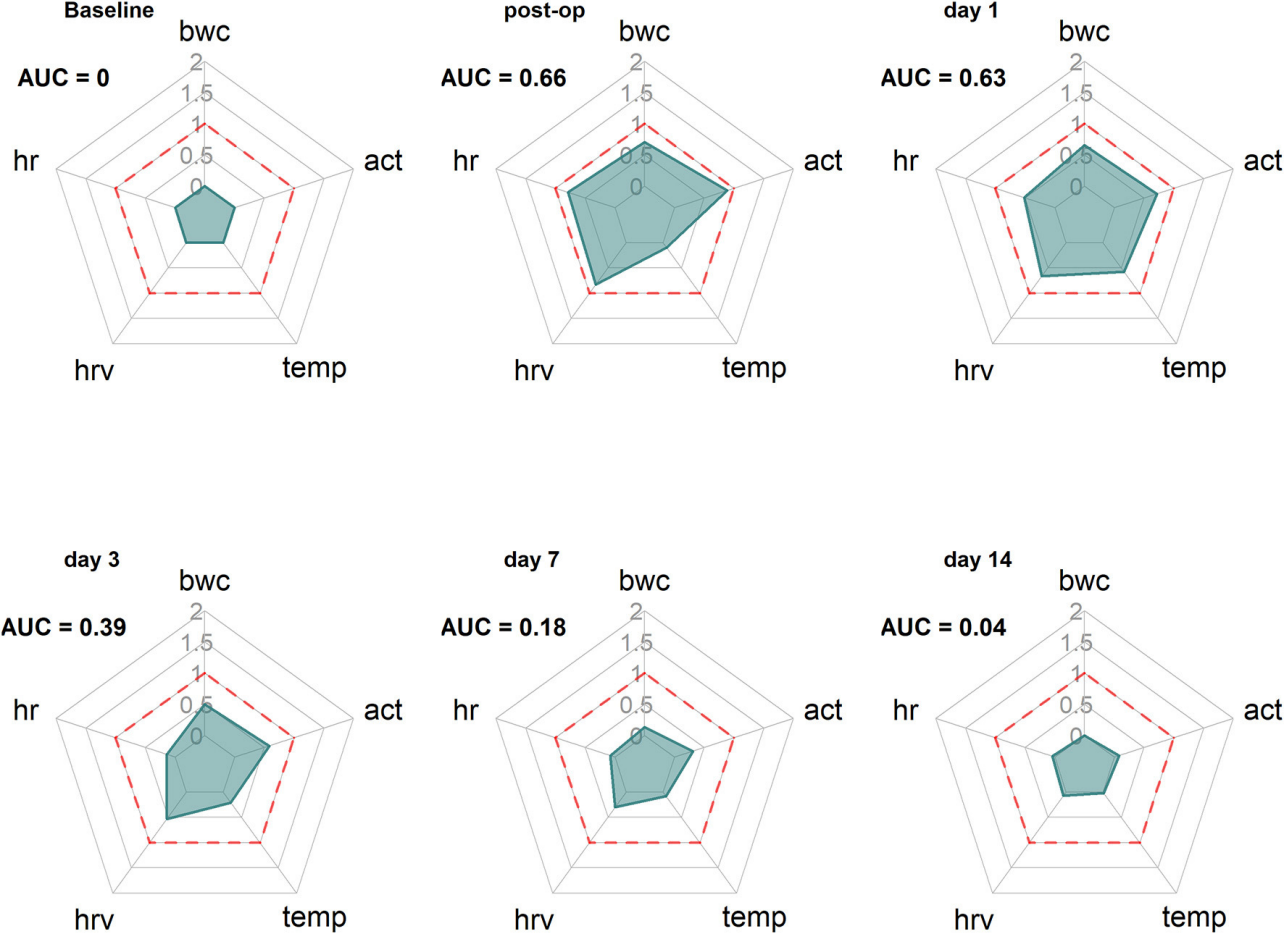
Radar charts reveal the time-dependent changing contribution of the outcome measures bwc, act, temp, hrv, and hr to the RELSA. The RELSA weights (RW) from the TM-implantation subgroup (*n* = 13) are averaged on six days (baseline, post-op day 0, day 1, day 3, day 7, and day 14) of the experiment. The dashed red line indicates the RELSA reference level of 1. Note, e.g., how the hrv stays elevated longer than the other outcome measures, showing the different and animal-model dependent qualities that single variables can assume in severity assessment.

The surgery data in this study showed that outcome measures varied in magnitude and showed differences concerning recovery times. In addition, the natural variance of biological systems is also part of any quantitative severity assessment [e.g., three individual animals in the Sham-group significantly deviated from the global RELSA mean (see Figure 4A)]. Therefore, to assess the contributions of individual outcome variables to the RELSA analysis, we monitored the average RELSA weight contributions of the surgery intervention [TM-implanted animals (Figure 7)] with radar charts.

The analysis of exemplary time points showed that the development in the variables changed over time. First, before the intervention (Baseline), the variables showed no contribution to the RELSA (AUC = 0). After surgery (post-op), all five variables showed substantial contributions (AUC = 0.66), e.g., the most notable contributor was the act variable with a weight contribution of RW_act,Bsl_ = 0.89. At the same time, temp contributed the least with RM_temp,Bsl_= 0.10 (Figure 7 Baseline). Finally, the contribution patterns changed over time, e.g., when the animals recovered. While all variables returned to their baseline positions, hrv and act remained more elevated than others.

The contribution patterns over time were animal-model specific. We also analyzed the additional studies for which RELSA analyses were performed. The RELSA performances of these studies are visualized in the Supplementary materials 8A–F. The corresponding radar charts/contribution patterns can also be found in the Supplemental material 9. Here, we saw, e.g., that the RELSA in the CLP model was dominated by the large differences in the temperature variable. However, the other outcome measures, except for bwc, also contributed but were not as strong as the temperature. Note that the time was reported in hours in the CLP study, not days (Supplementary materials 9.1–9.3). The sampling time or lag in bwc could not keep track of the fast changes in the severity status.

Interestingly, activity was the most contributing variable in CLP Sham animals (Supplementary material 9.3), but temperature and heart rate also contributed to the RELSA. Over the first days, the activity was the dominating variable in animals suffering from colitis with stress (Supplementary material 9.5) and colitis without stress (Supplementary material 9.4). Still, on day 7, body weight became more relevant. As expected, RELSA weights from colitis control mice showed only minor changes within any observed variables (Supplementary material 9.6).

## Discussion

More objective, comparable, evidence-based severity assessment methods are highly demanded. They offer a plethora of quality improvements regarding, e.g., (a) science, with higher standards in hypothesis testing, (b) the ability to monitor the best-possible individual animal welfare, (c) the ethical prerequisite for experimental refinement procedures, e.g., such as reducing the burdens in animals, and, (d) higher data quality, e.g., to counter the adverse effects of the reproducibility crisis. Finally, from a legal point of view, ensuring animal welfare and severity assessment is mandatory in many countries, e.g., in all EU member states (4). However, the large number and diversity of animal models and the lack of validated methods hinder clear definitions of severity categories (17). Consequently, this raises legal uncertainties for scientists and authorities, resulting in potential bias, e.g., in the actual and prospective severity ratings.

With the RELSA procedure, we addressed these critical points in laboratory animal science and developed a tool enabling an evidence-based severity assessment. RELSA uses an arbitrary number of outcome measures to compute a composite metric for welfare assessment and severity grading (18–20). To our knowledge, this is the first attempt in preclinical science to combine phenotypical data with the necessary experimental severity context to allow a qualitative grading between individual animals, subgroups, and models. This approach contrasts with current standards using human judgment to generate numerical scores for assessing welfare.

Addressing our initial hypothesis, we demonstrated that variables differed in performance and showed changing patterns in relative contributions over time, e.g., during the recovery phase. This empowers time-resolved refinement procedures, in which specific markers, e.g., for pain and temperature models, can be identified. Furthermore, the fact that these contribution patterns were highly animal-model-specific strengthens the concept of a multimodal severity assessment. Finally, RELSA paves the way for the field of comparative quantitative severity assessment, allowing the direct comparison of distinct animal models concerning severity levels. Ultimately, we speculate that the RELSA procedure will also apply to the human clinical context.

### RELSA in the current practice of composite scoring

The principle of composite scoring is based on systems utilized for clinical monitoring and risk assessment in human medicine. One example in humans is the Acute Physiology And Chronic Health Evaluation (APACHE II) score, first reported in 1985. The APACHE II score comprises 12 physiological and laboratory parameters with an additional weighting for age and preadmission health status to predict the risk of death (21, 22). In contrast, the Sequential Organ Failure Assessment (SOFA) score, established in 1996, consists of 6 different scores assessing distinct organ dysfunction and failure (23, 24). The score describes the status of morbidity and critical illness but does not predict the outcome. Currently, the SOFA score is used in the severity assessment of COVID-19 patients to characterize mortality among intensive care unit (ICU) patients (25). In veterinary medicine and laboratory animal science, there are various composite scores available, e.g., the clinical severity index for acute pancreatitis in canines (26), composite behavior scores for pain assessment in rodents (27, 28), or composite measure schemes for rat epilepsy models (9). These are elaborated systems tailored to model-specific characteristics which provide valuable insights into animal welfare.

To create a more generalized severity assessment approach that also addresses the specific needs of scientists and authorities working in laboratory animal science, we developed a procedure with the potential of combining any outcome measurement from clinical and behavioral examinations, thus widening the applicability across scientific fields. According to the EU directive, a severity classification is mandatory in the authorization process of animal experiments. However, the current classification poses several ambiguities as it is not comprehensive, not based on objective parameters, and does not consider refinement measures. Therefore, a comprehensive overview of classified models using evidence-based parameters will resolve this situation. RELSA provides a means to achieve this goal. From a scientific point of view, comparing the severity of different animal models on a multidimensional scale offers deep insight into the quantitative nature of animal wellbeing. This kind of severity mapping is a crucial feature that many in the 3R community request.

In addition, this approach considers the multidimensional nature of severity, reflecting pain and distress and affective, emotional states. We showed that not every variable reports the same severity information and that the content changes over time. Thus, the chosen parameters for severity assessment should be multimodal (12, 29). Furthermore, such a holistic approach enables refinement procedures. Multiple outcome variables indicate different sources of disturbed animal wellbeing over time, which is challenging or impossible to observe using single parameters. In addition, the RELSA procedure enables the comparison of models that differentially impact the welfare of animals on a relative scale. Of course, knowing these differences also allowed severity assessment in a well-understood and characterized model, using just the most prominent contributing variables. Therefore, when developing RELSA, we aimed at a quantitative grading of severity, while current methods in the veterinary sciences are characterized mainly by qualitative scorings.

### Outcome measures

In the present study, we used a comprehensive panel of methods to monitor the welfare of animals after various experimental procedures, e.g., with TM implantation as a use case. To exclude selection bias, we calculated the models' severity levels with a set of available outcome measures: body weight change, burrowing behavior, and telemetry-derived parameters, including hr, hrv, temperature, and activity. These outcome measures were selected based on increasing evidence of their suitability in various model systems as well as several round-table discussions within our German Research Foundation (DFG)-funded research consortium 2591, which focuses on severity assessment in animal-based research (www.severity-assessment.de) (7, 8, 29).

We observed that although some variables showed high sensitivity toward the implantation procedure, the change was short-lived. The most prominent example here is the burON variable. Burrowing is a highly motivated behavior of mice and is impaired under painful conditions or in mouse models of anxiety and schizophrenia (30, 31). In this study, burrowing was highly sensitive in detecting changes in welfare but only immediately after TM implantation. Likewise, bwc sensitively indicated the impact of TM surgery but quickly recovered within 4–6 days after the operation. Body weight is considered one of the most critical parameters in classic clinical scoring of rodents (32). However, monitoring body weight as a severity assessment parameter was model-specific and should be combined with other parameters (32).

In contrast, the telemetry-derived parameters hr, hrv, and act showed strong changes on the post-op day and indicated a longer-lasting impact on the animals, suggesting an extended recovery period (up to day 14). Telemetry is a frequently used method in biomedical research. For example, it has been shown that hr and hrv are suited for indicating distress and pain (33, 34), and hr and body temp serve as critical parameters in sepsis studies (35).

Our findings make us assume that the various parameters reflected different facets of severity (e.g., pain) better than others or that the animals lose some aspects over time. However, this exciting hypothesis remains elusive. The present results underpin the need for a combination of parameters to fully assess the (severity) situation, including physiological outcome measures. Therefore, the “usefulness” of outcome measures is dependent on the analytical purpose (e.g., acute pain vs. long-term impairment). We plan on expanding the RELSA applicability to this field.

### Using RELSA in comparisons

Usually, animals in a study are monitored over time, and the intervention effect is present somewhere on that timeline. However, in the TM-implantation model, the RELSA outcomes were skewed toward the time point with the most dominant deviations in the contributing outcome (post-op day). Since the exact maximum depends on the animal model under observation, a better choice for comparisons is the individual RELSA_max_ values representing each animal's time-independent maximally achieved RELSA values. The most extreme values reveal the maximally achieved severity better than the average. If the animal model is stable (e.g., showing consistent variance), the resulting RELSA_max_ values can be used, e.g., in animal model comparisons (Figure 6). Comparing the RELSA_max_ values revealed that TM implantation exhibited higher severity than Sham operations. However, the Sham operation also showed minimal severity due to natural variance.

### Validating the RELSA procedure

The RELSA procedure was validated using data from models with different forms and grades of impairments. In addition, data from an acute DSS-colitis model, an acute DSS colitis combined with repeated restraint stress, and a CLP sepsis model were assessed. Figure 6 shows that the RELSA_max_ values remained within the moderate frame of the four *k*-means cluster levels except for the CLP non-survivors and did not exceed the RELSA reference level of 1. In addition, the colitis RELSA_max_ values are reliably clustered in level L2, indicating a lower severity for the DSS-colitis model than in the TM-implantation study. However, 3 animals had to be euthanized in the colitis + stress study because the humane endpoint (max. of 20% weight loss) was reached. According to the project authorization, this was set to ensure that animals experience only a maximum of moderate severity levels. However, this also resulted in the loss of quantitative severity information. And although the RELSA values indicated increased suffering (Supplementary material 8D), they also implied that the animals might have been euthanized too soon, challenging the 20% loss in body weight threshold as an objective endpoint to ensure moderate severity levels. Even though the predefined endpoint in a single variable was reached, the remaining variables did not support a general increase in overall suffering concerning the reference set. Data from the CLP study revealed very high RELSA values in the animals that did not survive the procedure (RELSA_max_ ≥ 2.60) and lower values for the prevailing and Sham animals (RELSA_max_ < 1). The main factor responsible for the high values was the decrease in temperature, but hrv and act also indicated increases in severity. Here, more than one variable points toward increased suffering and an increased impairment in wellbeing. In addition to the between-model validation of the procedure, we validated RELSA internally for the reference data. Since we were not seeking to challenge established “gold-standard” procedures, we ensured that RELSA was at least comparable to or even better than clinical scoring (Supplementary material 7.1). Furthermore, the interval validation showed that RELSA is more precise with the current parameters and reveals information that subjective scoring could not catch (Supplementary material 7.2). Together with the model comparison capability of the RELSA, these features substantially improve the current standard of any severity assessment.

### RELSA principle and critical issues

RELSA enables scientists to quantify severity. The procedure can classify animals, subgroups, and animal models in a qualitative framework, e.g., mild, moderate, and severe. The necessary context must be provided as a reference set for such a qualitative grading. Ideally, this should be an animal model from which the qualitative severity context can be extrapolated while offering multiple outcome measures that consistently substantiate the quantitative scale. The caveat that makes up for the word “relative” in RELSA is that researchers must provide some qualitative estimate about the reference set's severity—ultimately, a step that still involves human judgment. However, once defined, a new experiment can be put into a quantitative severity context, always regarding the development in the reference set. This unique concept allows an evidence-based comparison of models within actual statutory provisions and guidelines. The Supplementary material explains the RELSA procedure so researchers can apply this method easily (Supplementary material 4).

In addition to providing context, the reference set has another purpose: it regularizes the possible ranges of the input variables. This can prove essential in severity assessment, as variables in negatively affected animals behave differently. For example, a loss of 17% in body weight is generally recognized as a threat to animal health (32). At the same time, the burrowing behavior may drop to zero. In this case, a difference of 17% in one variable is equivalent to a 100% difference in the other variable. For an optimal representation of this bias, we calculated individual RELSA weights (RW) as effect sizes for each variable and day, contributing to the final calculation. These weights can be considered a particular form of effect size somewhat related to Glass' Δ (36). However, for the RW values, the differences are that they are not standardized to the standard deviation in the control group but instead to the difference of the respective variable to its maximum deviation in the reference set. This approach estimates *within*-animal effect sizes and measurements of a particular variable's importance. We concluded that variables with larger deviations should have more impact on the generalization of the weights. In comparison, smaller deviations primarily represent noise and effects that are less prominent within a cohort. In statistics, this is followed by the root mean square (RMS) concept, e.g., in error and regression analysis. In contrast to a pure sum score, the RMS has the advantage that it directly translates to the scale of the individual weights and is considered more accurate in showing the best fit.

Another critical issue is the study-dependent sampling and measurement frequency of the outcome measure. For example, body weight is measured once per day (in the morning) and the burrowing behavior after a particular time (e.g., overnight). The sampling rates in these cases are (a) not equal and (b) not frequent enough to catch minute-by-minute changes. Transient changes in such variables thus appear as “all-or-nothing” parameters. Here, the biological changes happen faster than the sampling rates, so the exact development over time cannot be seen. Although the sampling rate cannot be corrected with RELSA, the skewness in distribution can be adjusted to a certain degree by including extreme values of a reference model with known severity in the calculation. This way, a model is backward compatible on the time scale, as we have shown with the CLP data, sampled on an hourly basis compared to the daily data in the reference model. To be comparable, we suggest that measurements in the reference set be from roughly the same reporting frame (e.g., day). This will also pave the way to a possible RELSA focusing on short-term bursts in severity changes (e.g., pain models) that were not covered in this study.

## Outlook and conclusion

RELSA was designed to assess the multidimensional severity facets that an animal experiences under impaired welfare conditions. Therefore, combining objective outcome measures into a composite metric has the advantage of an unbiased severity assessment without the need for interpretation or analysis. Furthermore, we have shown that such a hybrid model can be built, tested, and validated. In the future, comparing more animal models will lead to a severity map that can be used to better understand the multivariate nature of severity in laboratory animals. Finally, we have provided a framework that can be easily implemented into any severity-related research project's daily routine via the RELSA R package or web application. Eventually, assessing the severity and enabling the ranking of animal models in terms of their welfare impairment will become much more precise. This aspect may also reveal more generalized or specific variables for monitoring severity. With the development of home cage monitoring systems, RELSA will enable an automatic and continuous assessment of the animals and, thereby, an early warning system helping to identify animals at risk.

## Data availability statement

The datasets presented in this study can be found in online repositories. The names of the repository/repositories and accession number(s) can be found in the article/Supplementary material.

## Ethics statement

Experiments involving surgery, DSS colitis, and stress were reviewed and approved by the Local Institutional Animal Care and Research Advisory Committee and permitted by the Lower Saxony State Office for Consumer Protection and Food Safety (LAVES, Oldenburg, Lower Saxony, Germany; license 15/1905). The application for the animal experiments involving sepsis (authorization no. V54 – 19 c 20/15 - F152/1016) was approved by the local Ethics Committee for Animal Research (Darmstadt, Hessen, Germany). All procedures were carried out following the German law for animal protection and the European Directive 2010/63/EU.

## Author contributions

ST, CH, and AB conceptualized the study and drafted the manuscript. CH and AB designed the TM implantation and colitis study. CH, BS, LW, NW, MHe, LK, PJ, TK, MHo, and AK conducted the experiments, collected and annotated the data, and performed the descriptive analysis. ST redesigned the data annotations, developed, and coded the RELSA procedure, developed the R package and its applications, and conducted the corresponding (statistical) analyses. All authors discussed the results and commented on the manuscript.

## Conflict of interest

The authors declare that the research was conducted in the absence of any commercial or financial relationships that could be construed as a potential conflict of interest.

## Publisher's note

All claims expressed in this article are solely those of the authors and do not necessarily represent those of their affiliated organizations, or those of the publisher, the editors and the reviewers. Any product that may be evaluated in this article, or claim that may be made by its manufacturer, is not guaranteed or endorsed by the publisher.
