# Supplementary material for: RELSA—A multidimensional procedure for the comparative assessment of well-being and the quantitative determination of severity in experimental procedures
Source: Front Vet Sci. 2022 Nov 11;9:937711. doi: 10.3389/fvets.2022.937711 (PMC9691969; doi:10.3389/fvets.2022.937711)

Supplementary Material

S1 Animal numbers in the analyzed studies and subgroups.

Mouse model	Strain	Sex	n
TM implantation metamizole	C57BL/6J	♀	6
TM implantation carprofen	C57BL/6J	♀	7
sham surgery metamizole	C57BL/6J	♀	8
sham surgery carprofen	C57BL/6J	♀	7
CLP	C57BL/6N	♂	4
CLP sham	C57BL/6N	♂	3
DSS colitis	C57BL/6J	♀	8
DSS colitis+restraint stress	C57BL/6J	♀	7
Colitis control	C57BL/6J	♀	8

The raw data can be downloaded under the following link (available in the tab-delimited *.txt format). https://github.com/mytalbot/RELSA/tree/master/raw_data

S2 Expected tabular data format for RELSA analysis.

id	treatment	condition	time	bwc	hr	hrv	temp	act
Ca_001	Transmitter	Carprofen	-1	100.00	453.56	12.43	36.61	1064.84
Ca_001	Transmitter	Carprofen	0	87.50	660.59	2.05	36.18	125.54
Ca_001	Transmitter	Carprofen	1	90.76	584.92	4.84	37.50	407.34
…								

The data were taken from the example data in the RELSA online application. Note the baseline information given as time=-1. The table shows the first three entries of the non-normalized raw data (except bwc) of the TM-implantation study. The complete data can be downloaded and used in the corresponding application. https://calliope.shinyapps.io/RELSAapp/.

S3 RELSA R package

RELSA is available as an R-package with transparent code on GitHub. Installation notes and dependencies can be obtained here:

https://github.com/mytalbot/RELSA

Further, the package vignettes describing the procedure in detail and reproducible examples can be obtained from the RELSA website.

https://talbotsr.com/RELSA/

S4 A simple explanation of the RELSA procedure

RELSA is a straightforward procedure that merges the information of multiple variables into a single score representing the severity information of a sample concerning the maximum severity in a reference set. A RELSA value of 1 means that each input variable in a sample reached the utmost severity in the reference set. RELSA >1, therefore, indicates higher severity than the reference set.

Here, we present a simple example with three symbolic input variables: a) body weight change (%), b) burrowed material (%), and c) the change in heart rate (%) over time. All values are already standardized. This operation can be done with the RELSA package.

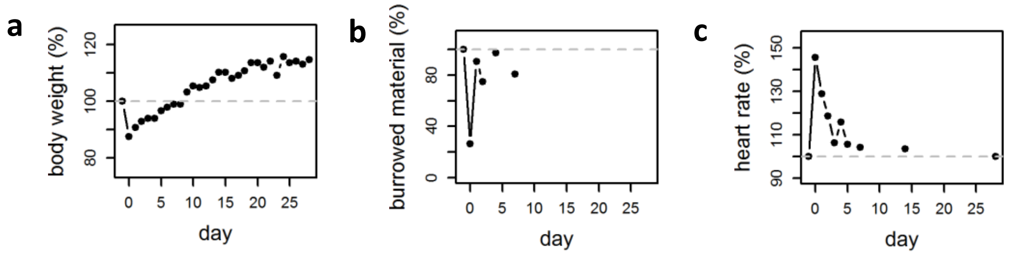

Note that there are missing data in some variables (e.g., in the burrowed material). The RELSA will always be calculated with the available information (e.g., just with body weight). While a drop in body weight indicates severity, e.g., in a surgery model, the opposite is the case with the heart rate. Here, an increase in heart rate will indicate severity. The directionality of this severity development in each variable is a necessary input for the RELSA algorithm. Since the directionality can change in unforeseeable ways in other animal models, it is not a viable option to calculate the absolute differences.

With the formula (1), the RELSA weights (RW) are calculated from each standardized value X_i. Since the baseline information is set to 100%, the RWs are defined as the difference of each variable to the baseline, divided by the maximum escalation of the same variable in the reference set.

$$RW = \frac{100 - X_i}{100 - \max(X_i)_{ref}} \quad (1)$$

This way, the RWs monitor the actual development of an input variable concerning the maximum changes (in terms of severity) in the reference data. The approach may result in large or small weight contributions. However, since small changes are less important than large changes (e.g., small changes may indicate noise), the usual mathematical approach to address this is the root mean square operation. Therefore, the final RELSA score is determined with formula (2). This operation can be performed on multiple time points to obtain RELSA curves.

$$RELSA = \sqrt{\frac{\sum_1^i RW}{\sum i}} \qquad (2)$$

Example 1

Let's assume a fictional reference set with the following maximum severity information: bwc=84.55%, burrowing=0%, and hr=153.39%. No measured values showed more extreme severity information during the experiment.

How much relative severity would an animal experience that had the following measurements in another (independent) experiment: bwc=87.5%, burrowing=26.46%, and hr=145.65%?

Table S4.1. Fictional RELSA calculation with three example variables resulting in RELSA<1.

	bwc	burrowing	hr
**Reference max**	84.55	0.00	153.39
**Testset (new study)**	87.50	26.46	145.65
**RELSA weights**	0.81	0.74	0.86
**RELSA**	0.80		

Table S4.1 shows the calculation of the RWs and the final RELSA score of 0.8 for the example animal. The RWs indicate that no variable exceeded the maximum in the reference set. Therefore, the result is correct, and the overall severity of that animal was lower. However, if we had additional qualitative knowledge, e.g., that the reference set was classified as "moderate" severity, we would know that the analyzed animal did not exceed the "moderate" severity status – in general or in any variable.

Example 2

The reference set remains the same in this example, but the measured values were more extreme than in example 1: bwc=81.1%, burrowing=2.0%, and heart rate 155.4%.

Table S4.2. Fictional RELSA calculation with three example variables resulting in RELSA>1.

	bwc	burrowing	hr
**Reference max**	84.55	0.00	153.39
**Testset (new study)**	81.10	2.00	155.40
**RELSA weights**	1.22	0.98	1.04
**RELSA**	1.09		

In table S4.2, the RWs for bwc and hr exceeded 1 as the values were larger than the ones in the reference set. On the other hand, the RW for burrowing was very close to 1 because the animals still burrowed 2% of the material. In total, this resulted in a RELSA >1 with 1.09. In this case, the animal would have experienced more relative severity than any animal in the reference set. Therefore, the quality of "moderate" severity would have been challenged.

S5 Cluster analysis of the three animal models

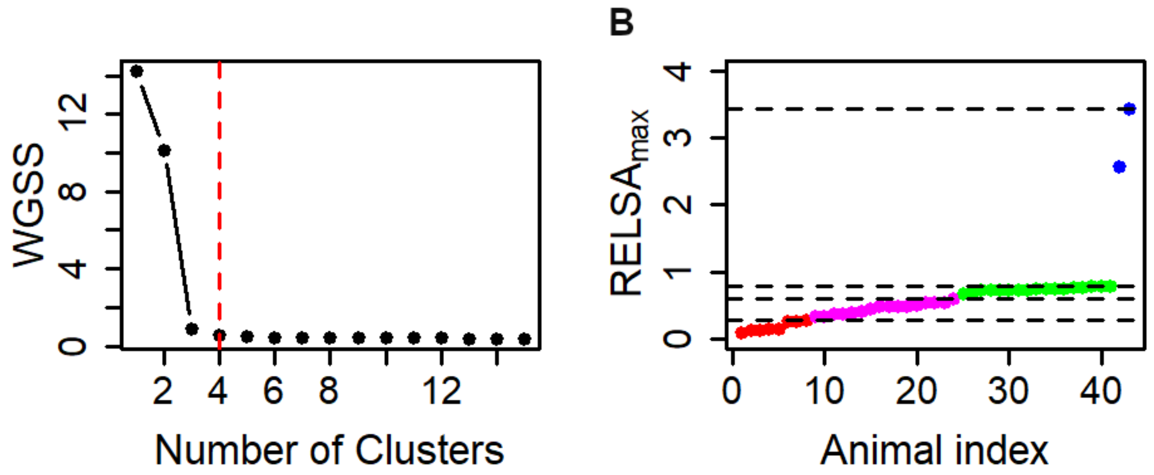

*(**A**) The heuristic Scree analysis of the number of clusters from a k-means analysis of the pooled RELSA data from three models (surgery, colitis, and sepsis) using bwc, hr, hrv, temp and act as input variables plotted against the within-groups sums of square (WGSS) results in k=4 clusters (red dashed line). (**B**) The k-means clustering of the RELSA$_{max}$ values translates to four cluster thresholds for the qualitative classification and grading of severity. Clusters were found at RELSA$_{max}$ levels: L1<0.27, L2<0.59, L3<0.79, and L4<3.45. The within-cluster data are color-coded.*

S6 Inferential analyses of the raw data

The analysis consists of three parts. The six outcome measures were analyzed separately and in the subgroups Transmitter implantation and Sham operation. For brevity, only significant results are shown. The following information is given:

a) The type III ANOVA table with interactions (treatment:day)
b) Multiple *between* treatment contrasts and comparisons/contrasts over time (with Tukey's posthoc test)[this analysis cannot be done with the TM data]
c) Baseline/day contrasts from an ANOVA with a control group (Baseline, BSL) followed by Dunnett's posthoc test

S6.1 Body Weight Change (%)

ANOVA Table.

	SS	Df	F	Pr(>F)
**(Intercept)**	150000	1	21852.55	<0.0001
**treatment**	0	1	0	1
**day**	8044.794	29	40.414	<0.0001
**treatment:day**	1645.071	29	8.264	<0.0001
**Residuals**	5306.017	773		

Daily between treatment contrasts (posthoc (Tukey) tests).

contrast	day	estimate	SE	Df	t-ratio	p_{adj}	stars
**Sham - Transmitter**	**0**	7.116	0.993	773	7.168	<0.0001	****
**Sham - Transmitter**	**1**	6.897	0.993	773	6.947	<0.0001	****
**Sham - Transmitter**	**2**	6.961	0.993	773	7.012	<0.0001	****
**Sham - Transmitter**	**3**	6.528	0.993	773	6.575	<0.0001	****
**Sham - Transmitter**	**4**	4.858	0.993	773	4.894	<0.0001	****
**Sham - Transmitter**	**5**	1.984	0.993	773	1.999	0.046	*
**Sham - Transmitter**	**6**	3.227	0.993	773	3.25	0.001	***
**Sham - Transmitter**	**7**	2.062	0.993	773	2.077	0.038	*

Baseline Comparisons (Dunnett test).

Transmitter						
contrast	estimate	SE	Df	t-ratio	p-value	stars
**0 - BSL**	-10.899	1.027	360	-10.609	<0.0001	****
**1 - BSL**	-10.075	1.027	360	-9.806	<0.0001	****
**2 - BSL**	-8.612	1.027	360	-8.382	<0.0001	****
**3 - BSL**	-7.723	1.027	360	-7.518	<0.0001	****
**4 - BSL**	-5.008	1.027	360	-4.874	<0.0001	****
**5 - BSL**	-3.38	1.027	360	-3.29	0.025	*
**10 - BSL**	3.205	1.027	360	3.12	0.042	*
**12 - BSL**	3.522	1.027	360	3.428	0.016	*
**13 - BSL**	5.305	1.027	360	5.164	<0.0001	****
**14 - BSL**	6.541	1.027	360	6.367	<0.0001	****
**15 - BSL**	7.233	1.027	360	7.04	<0.0001	****
**16 - BSL**	6.336	1.027	360	6.167	<0.0001	****
**17 - BSL**	6.917	1.027	360	6.733	<0.0001	****
**18 - BSL**	8.258	1.027	360	8.038	<0.0001	****
**19 - BSL**	9.197	1.027	360	8.953	<0.0001	****
**20 - BSL**	9.552	1.027	360	9.298	<0.0001	****
**21 - BSL**	9.346	1.027	360	9.097	<0.0001	****
**22 - BSL**	9.806	1.027	360	9.545	<0.0001	****
**23 - BSL**	9.597	1.027	360	9.341	<0.0001	****
**24 - BSL**	11.002	1.027	360	10.709	<0.0001	****
**25 - BSL**	10.689	1.027	360	10.405	<0.0001	****
**26 - BSL**	10.304	1.027	360	10.03	<0.0001	****
**27 - BSL**	10.324	1.027	360	10.049	<0.0001	****
**28 - BSL**	11.772	1.027	360	11.458	<0.0001	****

Baseline Comparisons (Dunnett test).

Sham						
contrast	estimate	SE	Df	t-ratio	p-value	stars
**0 - BSL**	-3.783	0.957	413	-3.953	0.002	**
**1 - BSL**	-3.178	0.957	413	-3.321	0.023	*
**12 - BSL**	3.55	0.957	413	3.71	0.006	**
**13 - BSL**	5.76	0.957	413	6.019	0	****
**14 - BSL**	5.991	0.957	413	6.261	0	****
**15 - BSL**	6.302	0.957	413	6.586	0	****
**16 - BSL**	6.532	0.957	413	6.826	0	****
**17 - BSL**	6.476	0.957	413	6.768	0	****
**18 - BSL**	7.34	0.957	413	7.671	0	****
**19 - BSL**	7.782	0.957	413	8.133	0	****
**20 - BSL**	7.62	0.957	413	7.963	0	****
**21 - BSL**	7.783	0.957	413	8.133	0	****
**22 - BSL**	8.001	0.974	413	8.216	0	****
**23 - BSL**	8.808	0.974	413	9.045	0	****
**24 - BSL**	9.058	0.974	413	9.302	0	****
**25 - BSL**	8.737	0.974	413	8.972	0	****
**26 - BSL**	9.792	0.974	413	10.055	0	****
**27 - BSL**	9.982	0.974	413	10.251	0	****
**28 - BSL**	10.559	0.974	413	10.843	0	****

S6.2 Transmitter variables

Baseline Comparisons (Dunnett test) - Transmitter Variables.

Heart Rate						
**contrast**	**estimate**	**SE**	**Df**	**t-ratio**	**p-value**	**stars**
**0 - BSL**	190.697	9.023	120	21.135	<0.0001	****
**1 - BSL**	124.706	9.023	120	13.821	<0.0001	****
**2 - BSL**	76.724	9.023	120	8.503	<0.0001	****
**3 - BSL**	31.933	9.023	120	3.539	0.005	**
**4 - BSL**	77.912	9.023	120	8.635	<0.0001	****
**5 - BSL**	24.974	9.023	120	2.768	0.047	*

Heart Rate Variability						
**contrast**	**estimate**	**SE**	**Df**	**t-ratio**	**p-value**	**stars**
**0 - BSL**	-10.967	0.753	120	-14.561	<0.0001	****
**1 - BSL**	-8.845	0.753	120	-11.744	<0.0001	****
**2 - BSL**	-7.494	0.753	120	-9.95	<0.0001	****
**3 - BSL**	-7.097	0.753	120	-9.422	<0.0001	****
**4 - BSL**	-7.116	0.753	120	-9.448	<0.0001	****
**5 - BSL**	-6.179	0.753	120	-8.204	<0.0001	****
**7 - BSL**	-4.023	0.753	120	-5.341	<0.0001	****

Temperature						
**contrast**	**estimate**	**SE**	**Df**	**t-ratio**	**p-value**	**stars**
**1 - BSL**	0.932	0.13	120	7.172	<0.0001	****
**2 - BSL**	0.763	0.13	120	5.87	<0.0001	****
**4 - BSL**	0.605	0.13	120	4.656	<0.0001	****

Activity						
**contrast**	**estimate**	**SE**	**Df**	**t-ratio**	**p-value**	**stars**
**0 - BSL**	-874.937	107.4	120	-8.146	<0.0001	****
**1 - BSL**	-731.694	107.4	120	-6.813	<0.0001	****
**2 - BSL**	-657.622	107.4	120	-6.123	<0.0001	****
**3 - BSL**	-603.86	107.4	120	-5.622	<0.0001	****
**4 - BSL**	-435.998	107.4	120	-4.06	0.001	***
**5 - BSL**	-383.455	107.4	120	-3.57	0.004	**
**7 - BSL**	-321.408	107.4	120	-2.993	0.025	*

S6.3 Burrowing over night

ANOVA Table.

	**SS**	**Df**	**F**	**Pr(>F)**
**(Intercept)**	130763.857	1	1144.5	<0.0001
**treatment**	42.661	1	0.373	0.542
**day**	1847.437	5	3.234	0.008
**treatment:day**	15957.73	5	27.933	<0.0001
**Residuals**	17823.942	156		

Daily between treatment contrasts (posthoc (Tukey) tests).

**contrast**	**day**	**estimate**	**SE**	**Df**	**t-ratio**	**p_{adj}**	**stars**
**Sham - Transmitter**	0	57.349	4.05	156	14.159	<0.0001	****
**Sham - Transmitter**	1	23.718	4.05	156	5.856	<0.0001	****

Baseline Comparisons (Dunnett test).

Transmitter						
**contrast**	**estimate**	**SE**	**Df**	**t-ratio**	**p-value**	**stars**
**0 - BSL**	-65.248	5.37	72	-12.15	<0.0001	****
**1 - BSL**	-19.465	5.37	72	-3.625	0.003	**

Baseline Comparisons (Dunnett test).

Sham						
**contrast**	**estimate**	**SE**	**Df**	**t-ratio**	**p-value**	**stars**
**0 - BSL**	-10.373	2.621	84	-3.958	0.001	***

S7 Internal RELSA validation

The individual RELSA scores were determined on each observation day using the following input variables: bwc, hr, hrv, temp, and act. In a subsequent analysis, the RELSA results were validated with a clinical score. Due to its subjective nature, the score was excluded from the RELSA calculations. In the first step of the validation process, the data of both analyses were averaged and visualized (Fig. S7.1) to compare the progression of severity. Next, the data were standardized to the range [1;0] and correlated (S7.2). Figure S7.2 shows the correlation of the clinical score with the RELSA results, fitted by linear regression.

S7.1 Qualitative validation of the RELSA score with the clinical score

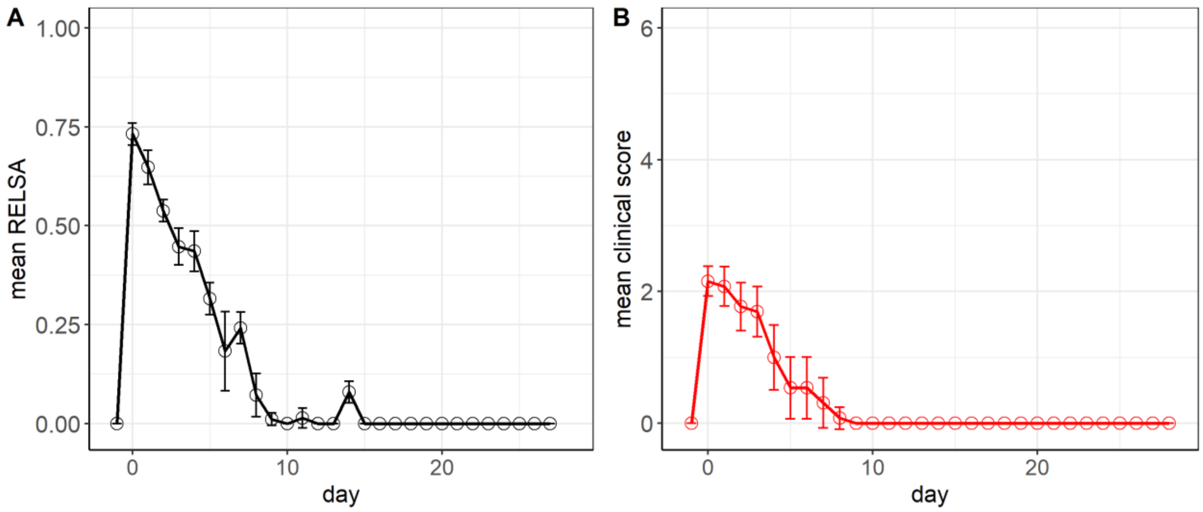

The time series in both (outcome) facets (S7.1 A and B) roughly show the same shape, e.g., with the largest deviation on day 0 (post-op day). However, the data are on different scales. The error bars are 95% confidence intervals. After surgery, the transmitter-implanted animals (n=13) were scored daily for 7 days and afterward every 3 to 4 days using a score adapted from Morton and Griffiths (Morton, DB, Griffiths, PHM. 1985. Guidelines on the recognition of pain, distress, and discomfort in experimental animals and a hypothesis for assessment. Vet Rec 116: 431– 436). The score included the monitoring of the body weight, as well as the visual evaluation of the activity, general health condition, and behavior. Score values ranging from 0 (no impairment) to 6+ (severe impairment) were assigned. In this case, the precision of the clinical score was drastically lower than in the objectively measured variables (i.e., the standardized median ranges of the error bars in the score data were 3.37-times larger than in the RELSA). Nevertheless, the averaged and scaled measures were highly correlated (r=0.98, $CI_{95\%}$[0.95; 0.99], t = 22.81, df = 27, p-value < 0.0001), which validated the general function of the RELSA algorithm for the reference set.

S7.2 The linear fit of the non-averaged RELSA and clinical score values shows the advantage of the RELSA procedure

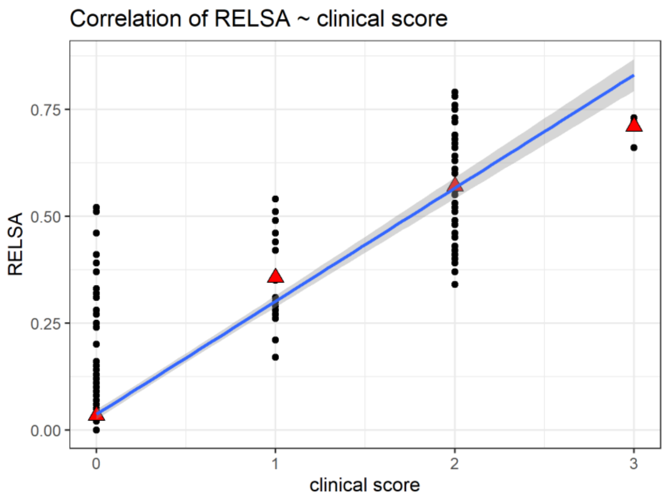

On the non-averaged scale, the clinical score and the RELSA are highly correlated but show a more considerable variance. The linear regression is significant, but the fit is slightly worse than the averaged data ($F(3,359)= 506.7$, $p<0.0001$, $R^2_{adj}= 0.81$). The red triangles represent the average RELSA values per score category. Note some higher RELSA values at score=0. These outcomes indicate potential severity information, which may be challenging to observe with the clinical score but was detected using the RELSA method.

Interestingly, some RELSA values at score = 0 appear inefficiently high, although the average was near zero ($RELSA_0= 0.03$). When looking at the RELSA weights, it can be seen that these data include severity information from hrv, act, and temp but only in small proportions. These measures may point to severity information that is difficult to assess with the clinical score (e.g., heart rate variability). Looking at these entries with score=0 but RELSA ≠ 0 reveals 51 entries for which the RELSA weights can be averaged (Table S7.3).

Table S7.3. Averaged RELSA weight information from score=0 & RELSA ≠ 0 cases.

bwc	hr	hrv	temp	act
0.08	0.14	0.30	0.13	0.27

The main components of severity represented by these cases originate in hrv (RW=0.3) and activity (RW=0.27) information. Both variables are not well represented in the clinical score or may even be challenging to assess by a human observer. In the case of the variables obtained by transmitter implantation, the RELSA method provides this hidden severity information and, therefore, has advantages over the traditional clinical scoring method. However, this outcome may depend on the choice of variables.

Please note that even though the RELSA procedure is shown with telemetry data, researchers are not bound to use these variables. The RELSA pipeline can be built with any other variables to fit the individual need of research projects. However, utilizing multiple objectively measured variables to obtain the complete severity picture in each animal is highly recommended to avoid subjective bias. While the RELSA procedure has advantages over clinical scoring, its true power lies in model comparability using multiple input variables.

S8 RELSA performance of the CLP and colitis study subgroups

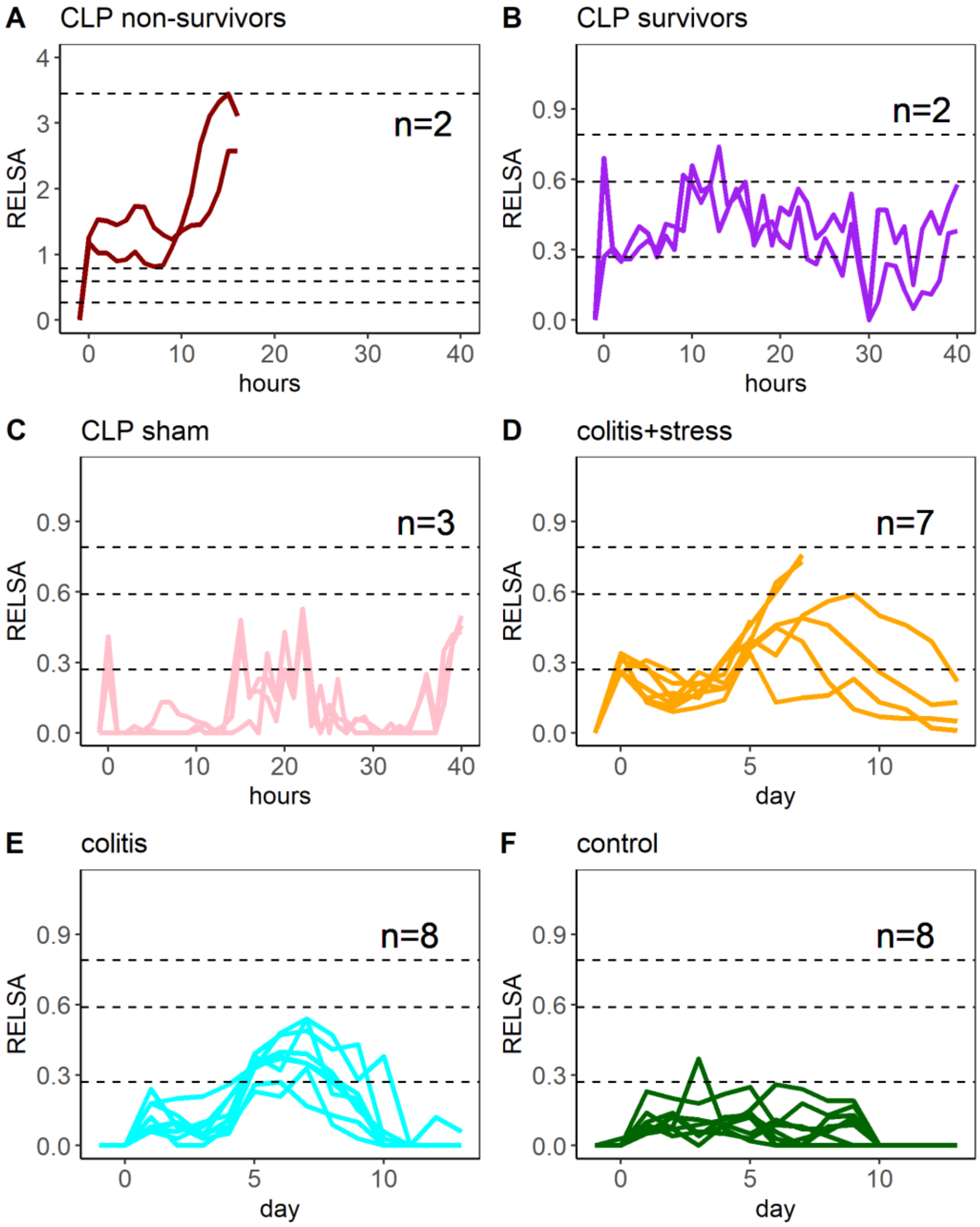

*(**A**) Time-dependent RELSA outcomes of animals in the cecal ligation puncture (CLP) study, including the CLP non-survivors (brown lines), (**B**) the CLP survivors (purple lines) and (**C**) the CLP sham-operated animals (pink lines). The time variable in the CLP study is given in hours, not days, as in the other studies. (**D**) The RELSA development in the mice from the colitis+stress study (yellow lines) shows three broken lines representing three euthanized animals due to meeting the endpoint criterion of 20% body weight loss. (**E**) The turquoise lines represent mice suffering from colitis without additional stress treatment, and (**F**) RELSA values from the corresponding control animals are shown with the green lines. The dashed lines represent the four severity thresholds from a k-means clustering of the surgery RELSA reference model (L1<0.27, L2<0.59, L3<0.79, and L4<3.45) to enable a comparative grading and categorization of the models and animals.*

S9 Radar Charts of the additionally analyzed studies

S9.1 CLP non-survivors

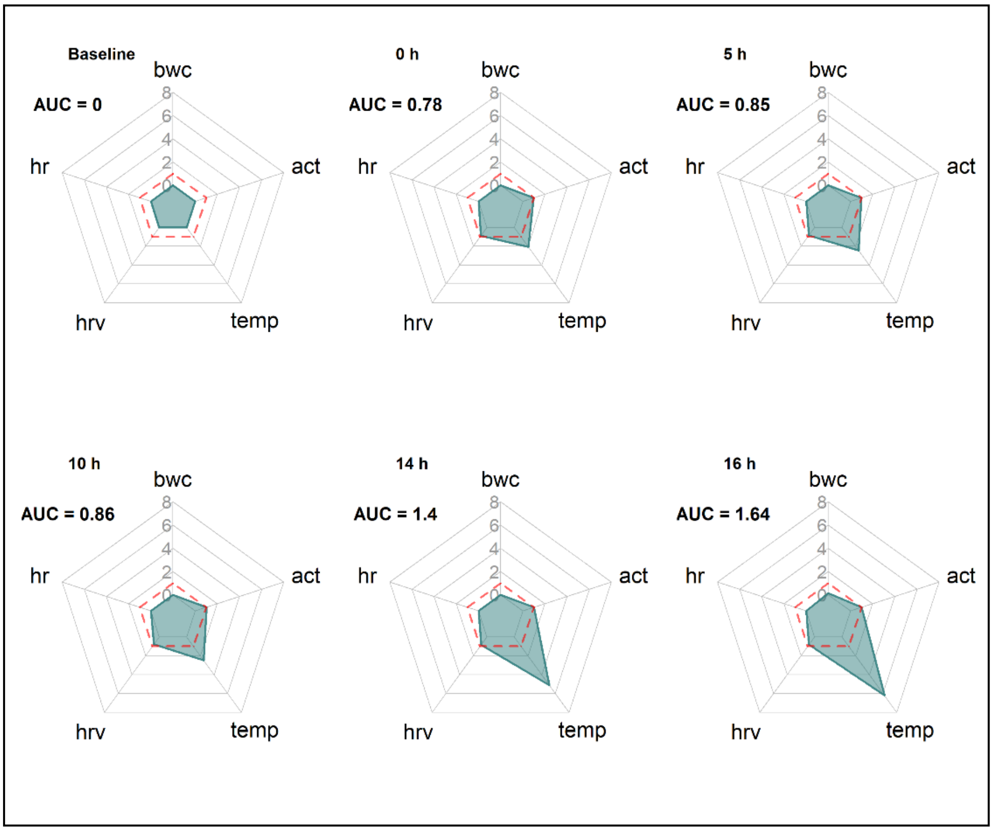

S9.2 CLP survivors

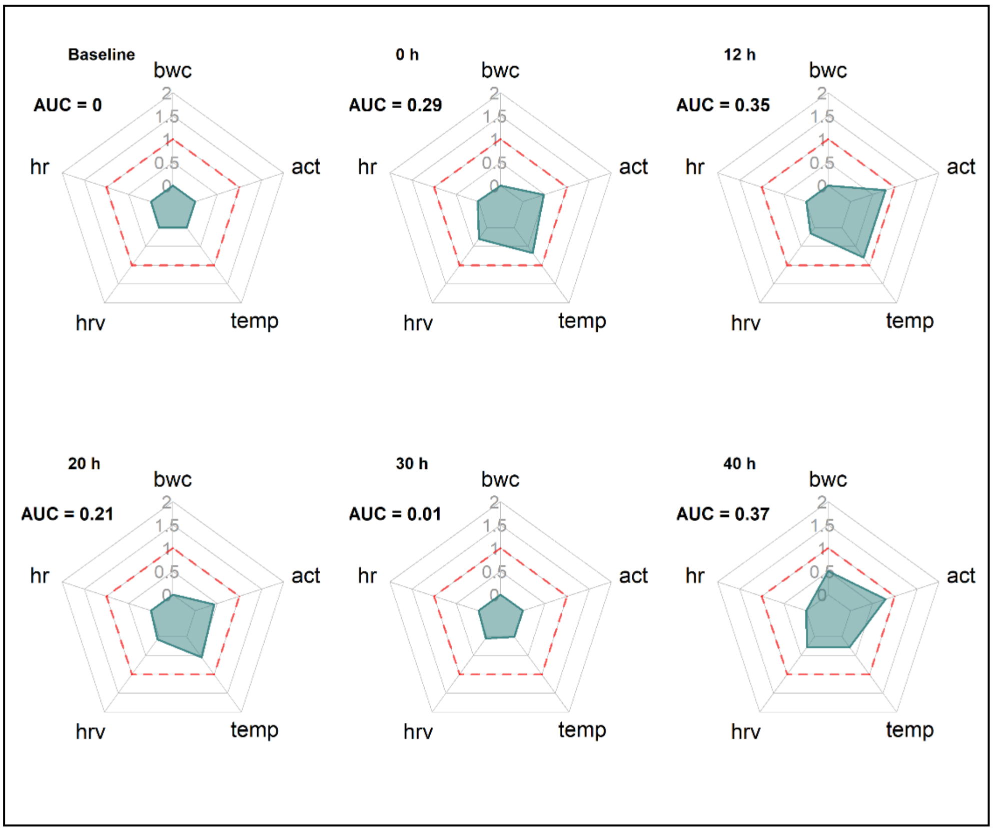

S9.3 CLP sham

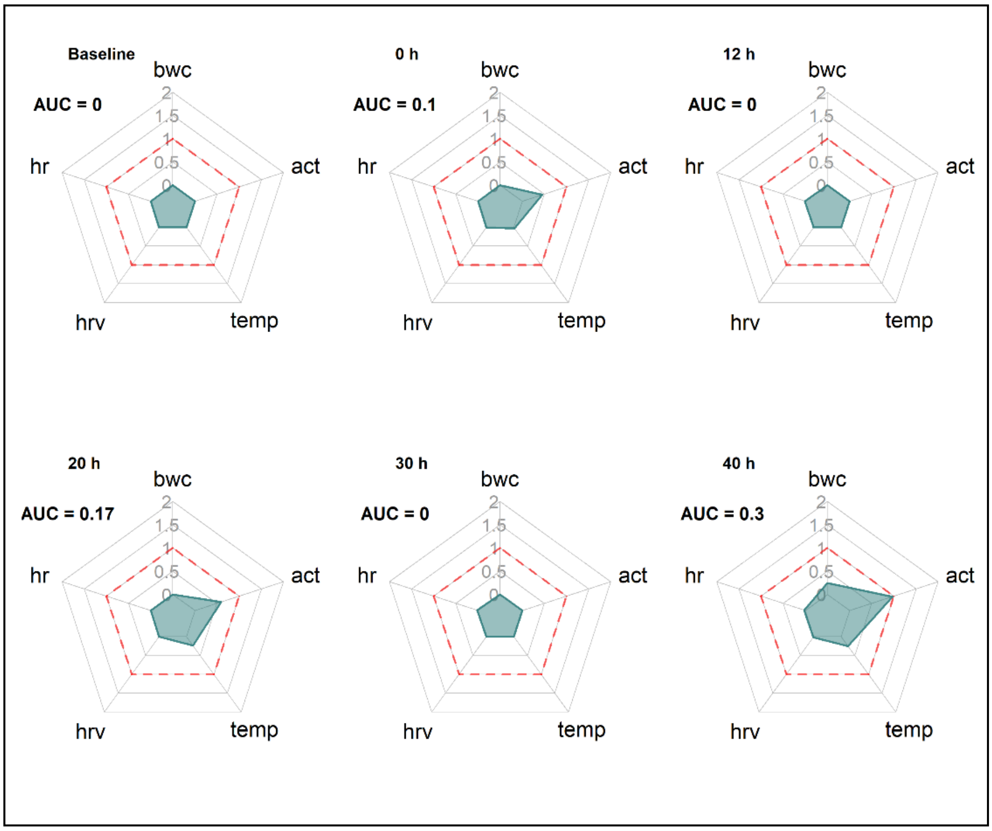

S9.4 Colitis

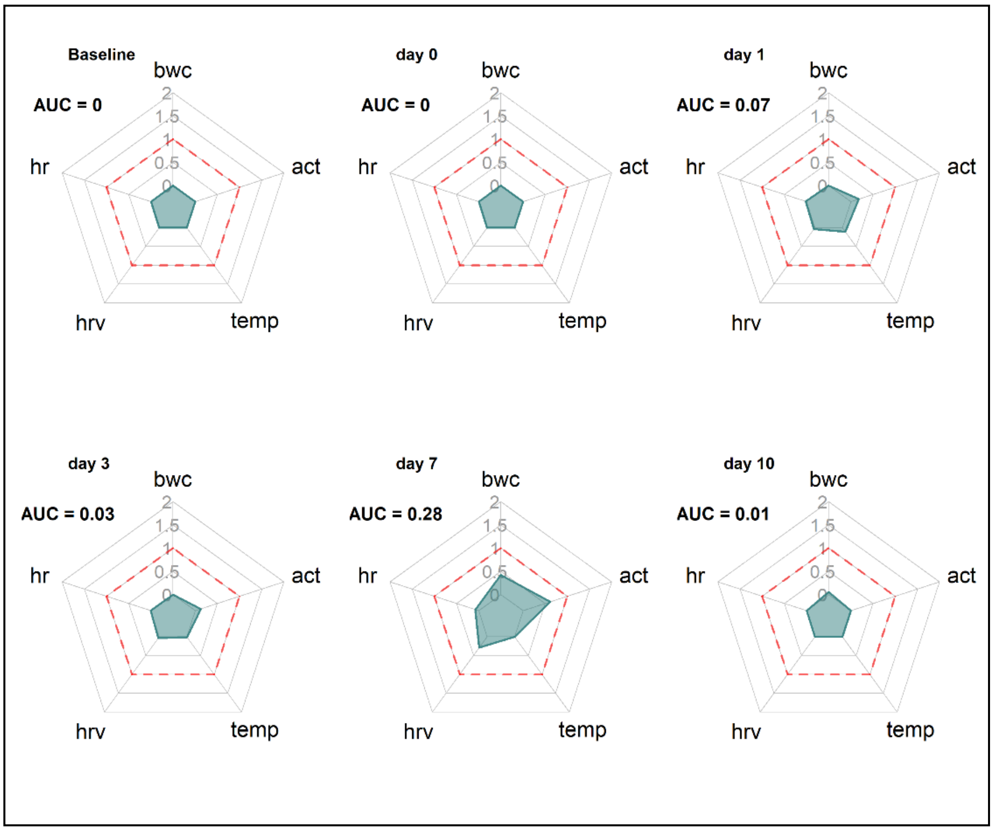

S9.5 Colitis + Stress

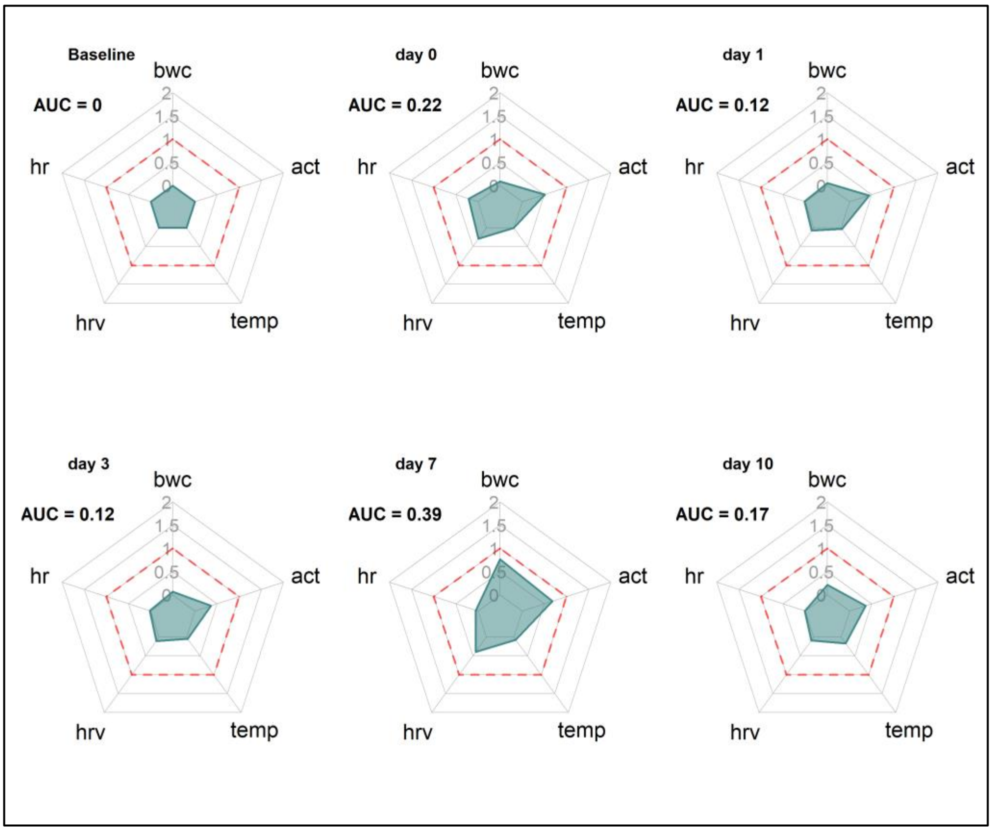

S9.6 Colitis control

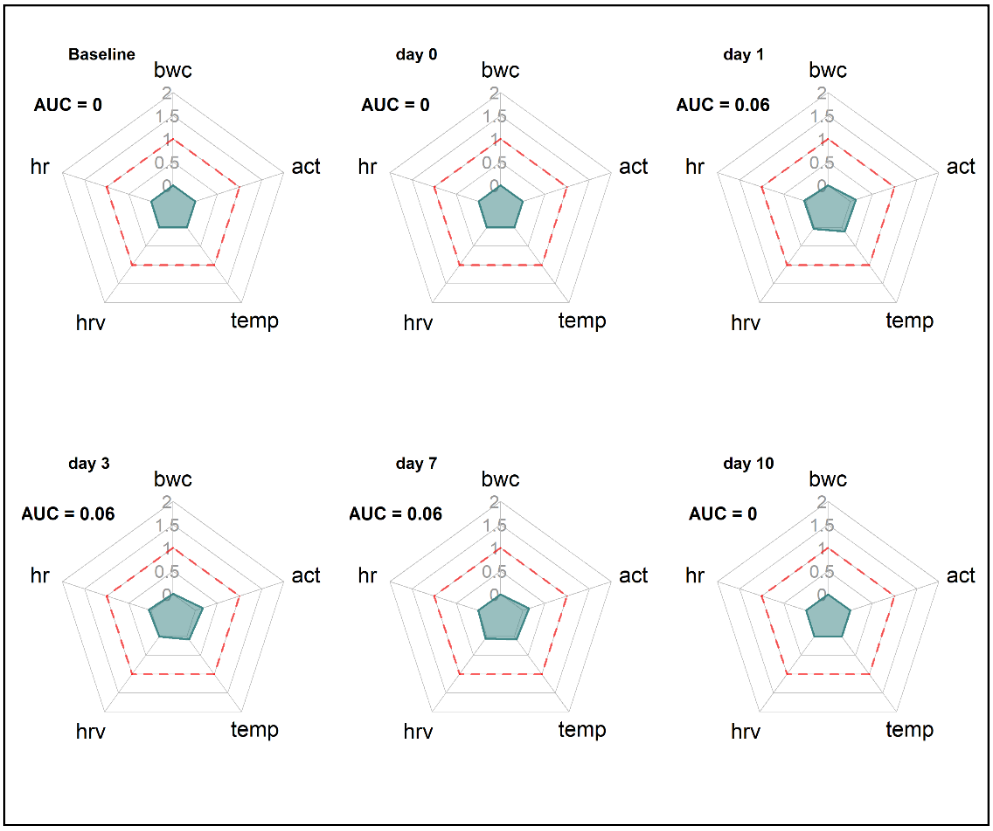

Supplement: Supplementary file 1 [file Data_Sheet_1.pdf]
